# Cross-cohort gut microbiome associations with immune checkpoint inhibitor response in advanced melanoma

**DOI:** 10.1038/s41591-022-01695-5

**Published:** 2022-02-28

**Authors:** Karla A. Lee, Andrew Maltez Thomas, Laura A. Bolte, Johannes R. Björk, Laura Kist de Ruijter, Federica Armanini, Francesco Asnicar, Aitor Blanco-Miguez, Ruth Board, Neus Calbet-Llopart, Lisa Derosa, Nathalie Dhomen, Kelly Brooks, Mark Harland, Mark Harries, Emily R. Leeming, Paul Lorigan, Paolo Manghi, Richard Marais, Julia Newton-Bishop, Luigi Nezi, Federica Pinto, Miriam Potrony, Susana Puig, Patricio Serra-Bellver, Heather M. Shaw, Sabrina Tamburini, Sara Valpione, Amrita Vijay, Levi Waldron, Laurence Zitvogel, Moreno Zolfo, Elisabeth G. E. de Vries, Paul Nathan, Rudolf S. N. Fehrmann, Véronique Bataille, Geke A. P. Hospers, Tim D. Spector, Rinse K. Weersma, Nicola Segata

**Affiliations:** 1 Department of Twin Research and Genetic Epidemiology, King’s College London, London, UK; 2 Department CIBIO, University of Trento, Trento, Italy; 3 Department of Gastroenterology and Hepatology, University of Groningen and University Medical Center Groningen, Groningen, the Netherlands; 4 Department of Medical Oncology, University of Groningen and University Medical Center Groningen, Groningen, the Netherlands; 5 Department of Oncology, Lancashire Teaching Hospitals NHS Trust, Preston, UK; 6 Dermatology Department, Hospital Clínic Barcelona, Universitat de Barcelona, IDIBAPS, Barcelona, Spain; 7 Centro de Investigación Biomédica en Red en Enfermedades Raras, Instituto de Salud Carlos III, Barcelona, Spain; 8 U1015 INSERM, University Paris Saclay, Gustave Roussy Cancer Center and Oncobiome Network, Villejuif-Grand-Paris, France; 9 Molecular Oncology Group, CRUK Manchester Institute, University of Manchester, Manchester, UK; 10 Division of Haematology and Immunology, Institute of Medical Research at St. James’s, University of Leeds, Leeds, UK; 11 Biochemical and Molecular Genetics Department, Hospital Clínic de Barcelona, IDIBAPS and University of Barcelona, Barcelona, Spain; 12 Department of Medical Oncology, Guys Cancer Centre, Guys and St Thomas’s NHS Trust, London, UK; 13 The Christie NHS Foundation Trust, Manchester, UK; 14 Division of Cancer Sciences, University of Manchester, Manchester, UK; 15 European Institute of Oncology (Istituto Europeo di Oncologia, IRCSS), Milan, Italy; 16 Department of Medical Oncology, Mount Vernon Cancer Centre, Northwood, UK; 17 Rheumatology & Orthopaedics Division, School of Medicine, University of Nottingham, Nottingham, UK; 18 Graduate School of Public Health and Health Policy, City University of New York, New York, NY USA; 19 Department of Dermatology, Mount Vernon Cancer Centre, Northwood, UK

**Keywords:** Prognostic markers, Metagenomics

## Abstract

The composition of the gut microbiome has been associated with clinical responses to immune checkpoint inhibitor (ICI) treatment, but there is limited consensus on the specific microbiome characteristics linked to the clinical benefits of ICIs. We performed shotgun metagenomic sequencing of stool samples collected before ICI initiation from five observational cohorts recruiting ICI-naive patients with advanced cutaneous melanoma (*n* = 165). Integrating the dataset with 147 metagenomic samples from previously published studies, we found that the gut microbiome has a relevant, but cohort-dependent, association with the response to ICIs. A machine learning analysis confirmed the link between the microbiome and overall response rates (ORRs) and progression-free survival (PFS) with ICIs but also revealed limited reproducibility of microbiome-based signatures across cohorts. Accordingly, a panel of species, including *Bifidobacterium pseudocatenulatum*, *Roseburia* spp. and *Akkermansia muciniphila*, associated with responders was identified, but no single species could be regarded as a fully consistent biomarker across studies. Overall, the role of the human gut microbiome in ICI response appears more complex than previously thought, extending beyond differing microbial species simply present or absent in responders and nonresponders. Future studies should adopt larger sample sizes and take into account the complex interplay of clinical factors with the gut microbiome over the treatment course.

## Abstract (web summary)

An analysis of metagenomic sequencing of stool samples from multiple cohorts of patients with melanoma treated with immune checkpoint blockade uncovers microbiome correlates of response to therapy and also reveals widespread variability across populations.

## Main

Therapeutic targeting of immune checkpoints such as programmed death-ligand 1, programmed cell death protein 1 (PD-1) and cytotoxic T-lymphocyte-associated protein 4 (CTLA-4) with ICIs has revolutionized the treatment of advanced melanoma. Several landmark randomized controlled trials have shown notable and durable survival benefits, resulting in changes to standard of care internationally^1,2^. Presently, over 50% of patients treated with a combination of PD-1 and CTLA-4 blockade are alive after five years^1^. Despite these advances, fewer than half of the patients who receive a single-agent ICI respond to it, whereas a higher response to combined targeting of PD-1 and CTLA-4 is associated with frequent toxicity with immune-related adverse events^3,4^.

The discovery of a link between the gut microbiome and response to ICIs, in melanoma and other tumors, highlighted the gut microbiome as a potential biomarker of response^5,6^ as well as a therapeutic target^7,8^. Although there is compelling evidence for specific gut microbial features associated with beneficial responses in mouse studies^9,10^, little consensus exists on which microbiome characteristics are associated with treatment responses in the human setting. In one of the largest metagenomic studies to date, Routy et al.^11^ found responders to harbor significantly higher relative abundances of *Akkermansia muciniphila*, *Alistipes* and generally more *Firmicutes* compared to nonresponders^11^, whereas Gopalakrishnan et al.^6^ found a higher relative abundance of *Faecalibacterium prausnitzii* in responders compared with nonresponders. In addition, Matson et al.^5^ found that responsiveness to PD-1 therapy was defined by an increased relative abundance of a group of eight species driven by *Bifidobacterium longum*. Frankel et al. reported that microbiota differed by ICI regimen but that enrichment of *Bacteroides caccae* was common in responders treated with any ICI regimen^12^. Several confounding factors may have contributed to this lack of consensus, such as collection and DNA extraction protocols, dietary and medication-use differences among countries, issues of sample size and statistical power, variability in microbiome signatures among responders and microbial signals that are functionally related but intrinsic to each cohort. Cohort effects ranging from population-specific characteristics to methodological choices in sample processing and analysis are important problems in microbiome studies^13,14^. Thus, larger and diverse cohorts with metagenomic data and standardized metadata are needed to better elucidate the microbiome determinants of response to immunotherapy.

The Predicting Response to Immunotherapy for Melanona with Gut Microbiome and Metabolomics (PRIMM) studies are two separate prospective observational cohort studies recruiting subjects in parallel in the United Kingdom (PRIMM-UK) and the Netherlands (PRIMM-NL) since 2018. These cohorts of previously ICI-naive patients with advanced melanoma provide extensive biosamples, including stool, serum and peripheral blood mononuclear cells, before and during ICI treatment, with detailed clinical and dietary data collected at regular intervals longitudinally. To study the role of the gut microbiome in ICI response, we performed shotgun metagenomic sequencing of baseline stool samples from the PRIMM cohorts, as well as three additional cohorts of ICI-naive patients with advanced cutaneous melanoma (originating from Barcelona, Leeds and Manchester). A total of 165 microbiome samples sequenced from the patients enrolled in this study were analyzed together with 147 samples from smaller publicly available datasets. This method provided the largest assessment of the potential of the gut microbiome as a biomarker of response to ICI and allowed for investigation of specific microbial species or functions associated with response.

## Results

In total, we recruited 175 patients from five distinct cohorts across the United Kingdom, the Netherlands and Spain with unresectable stage III and stage IV cutaneous melanoma who received ICI treatment after collecting stool samples (Table 1). We initially focused on the two single prospectively recruited PRIMM cohorts with more than 50 samples each, both of which exceed the sample size of all previously published datasets. In these cohorts, comprehensive clinical data, standardized radiological response assessments (RECIST v1.1) and biological specimens were collected before treatment and longitudinally at each cycle of treatment. There were statistically relevant clinical differences between these two cohorts, including the proportion of subjects who had received previous systemic targeted therapy (40% within PRIMM-NL and 20% within PRIMM-UK, *P* = 0.03, Fisher’s exact test), the proportion with BRAF-mutated tumors (55% within PRIMM-NL and 31% within PRIMM-UK, *P* = 0.02, Fisher’s exact test), the proportion of patients receiving combination ICI (ipilimumab and nivolumab) (22% within PRIMM-NL and 53% within PRIMM-UK, *P* = 0.003, Fisher’s exact test, Supplementary Table 1) and the proportion of patients with M1d disease (36% within PRIMM-NL and 9% within PRIMM-UK, *P* = 0.0005, Fisher’s exact test). For these reasons, we decided to analyze the cohorts separately. All samples were subjected to shotgun metagenomic sequencing at an average sequencing depth of 7.74 Gb, and the 165 samples that passed strict quality control were analyzed at the taxonomic and functional potential levels using bioBakery 3 (ref. ^15^).

**Table 1:** Patient characteristics at study entry, treatment details and outcomes by RECIST v1.1

	PRIMM-UK (*n* = 55)	PRIMM-NL (*n* = 55)	Manchester (*n* = 25)	Leeds (*n* = 18)	Barcelona (*n* = 12)	All cohorts (*n* = 165)
Gender, *n* (%)						
Male	36 (65%)	31 (56%)	16 (64%)	11 (61%)	6 (50%)	100 (61%)
Female	19 (35%)	24 (44%)	9 (36%)	7 (39%)	6 (50%)	65 (39%)
Age (yr) at stage IV diagnosis, median (range)	65 (19-94)	61 (25-85)	66 (36-87)	60 (35-88)	64 (37-88)	63 (19-94)
Metastatic stage						
Stage 3 unresectable	5 (9%)	1 (2%)	0 (0%)	1 (6%)	0 (0%)	7 (4%)
M1a	13 (24%)	7 (13%)	5 (20%)	3 (17%)	4 (33%)	32 (20%)
M1b	12 (22%)	9 (16%)	6 (24%)	5 (27%)	5 (42%)	37 (22%)
M1c	20 (36%)	18 (33%)	11 (44%)	6 (33%)	3 (25%)	58 (35%)
M1d	5 (9%)	20 (36%)	3 (12%)	3 (17%)	0 (0%)	31 (19%)
BRAF mutant, *n* (%)	17 (31%)	30 (55%)	3 (12%)	8 (44%)	3 (25%)	61 (37%)
Previous systemic antimelanoma therapy, *n* (%)	11 (20%)	22 (40%)	7 (28%)	0 (0%)	1 (8%)	41 (25%)
Performance status						
0	17 (31%)	36 (65%)	15 (60%)	16 (89%)	7 (58%)	91 (55%)
1	32 (58%)	12 (22%)	9 (36%)	2 (11%)	1 (8%)	56 (34%)
2	5 (9%)	4 (7%)	0 (0%)	0 (0%)	0 (0%)	9 (5%)
3	1 (2%)	1 (2%)	1 (4%)	0 (0%)	0 (0%)	3 (2%)
Unknown	0 (0%)	2 (4%)	0 (0%)	0 (0%)	4 (34%)	6 (4%)
BMI (kg m^−2^), mean (range)	28.7 (18.8–47.7)	27.3 (18.8–40.7)	26.9 (19.0–35.8)	30.2 (21.6–38.6)	27.1 (21.0–36.1)	28 (18.8–47.7)
Antibiotic use within 3 months of ICI, *n* (%)	9 (16%)	11 (20%)	4 (16%)	2 (11%)	0 (0%)	26 (16%)
PPI use within 3 months of ICI, *n* (%)	14 (25%)	20 (36%)	4 (16%)	5 (28%)	1 (8%)	44 (27%)
Treatment details						
ICI used						
Ipilimumab and nivolumab	29 (53%)	12 (22%)	2 (8%)	10 (56%)	1 (8%)	54 (32%)
Pembrolizumab	18 (33%)	10 (18%)	13 (52%)	3 (17%)	9 (75%)	53 (37%)
Nivolumab	8 (14%)	32 (58%)	0 (0%)	5 (27%)	2 (17%)	48 (24%)
Ipilimumab	0 (0%)	1 (2%)	10 (40%)	0 (0%)	0 (0%)	11 (7%)
Outcomes following ICI						
RECIST v1.1 response at 6 months						
Complete response	8 (15%)	6 (11%)	4 (16%)	2 (12%)	2 (17%)	22 (13%)
Partial response	16 (29%)	16 (29%)	6 (24%)	4 (22%)	1 (8%)	43 (26%)
Stable disease	9 (16%)	11 (20%)	3 (52%)	4 (22%)	2 (17%)	29 (18%)
Progressive disease	22 (40%)	22 (40%)	12 (48%)	8 (44%)	7 (58%)	71 (43%)
PFS12, *n* (%)	28 (51%)	30 (55%)	13 (52%)	8 (44%)	7 (58%)	86 (52%)

BMI, body mass index; PFS12, PFS at 12 months; PPI, proton pump inhibitor.

### Linking the gut microbiome with response in the PRIMM cohorts

**Fig. 1:**
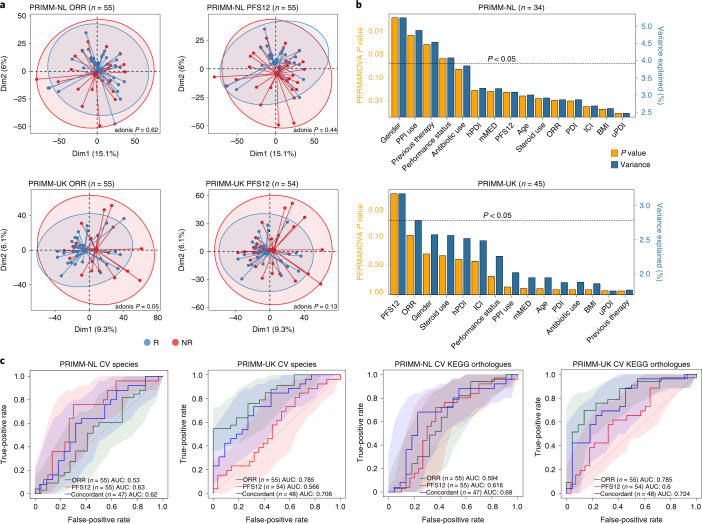
Association between the gut microbiome and response in the PRIMM-NL and PRIMM-UK cohorts. **a**, Response evaluated by ORR (Methods) is associated with the overall microbiome structure for PRIMM-UK (*P* = 0.05), but not for PRIMM-NL or PFS12, as represented visually using principal-component analysis (PCA) of species-level centered log-ratio-transformed relative abundances. *P* values were calculated using adonis and 999 permutations (Extended Data Figs. 2 and 3 show additional beta diversity analysis). Dim1, dimension 1; Dim2, dimension 2. **b**, Multivariate analysis showing the amount of inferred variance explained (*R*^2^, blue vertical bars) by each identified covariate and their respective *P* value (orange vertical bars) as determined by PERMANOVA on species-level centered log-ratio-transformed relative abundances. **c**, Machine learning association analysis between taxonomic (species abundance) and functional profiles (KEGG ortholog abundances) of the microbiome and response showed consistent associations with both response types (ORR or PFS12). The ‘concordant’ label includes only patients who did not progress between 6 and 12 months. AUC-ROC curves are computed using Lasso models trained using 100-repeated fivefold-stratified cross-validations. Shaded areas represent AUC-ROCs from each individual machine learning model. ICI indicates the use of a combination of ipilimumab and nivolumab or single agent. AUC, area under the curve; CV, cross-validation; hPDI, healthy plant-based diet index; PDI, plant-based diet index; uPDI, unhealthy plant-based diet index; mMED, modified Mediterranean diet score.

**Extended Data Fig. 1:**
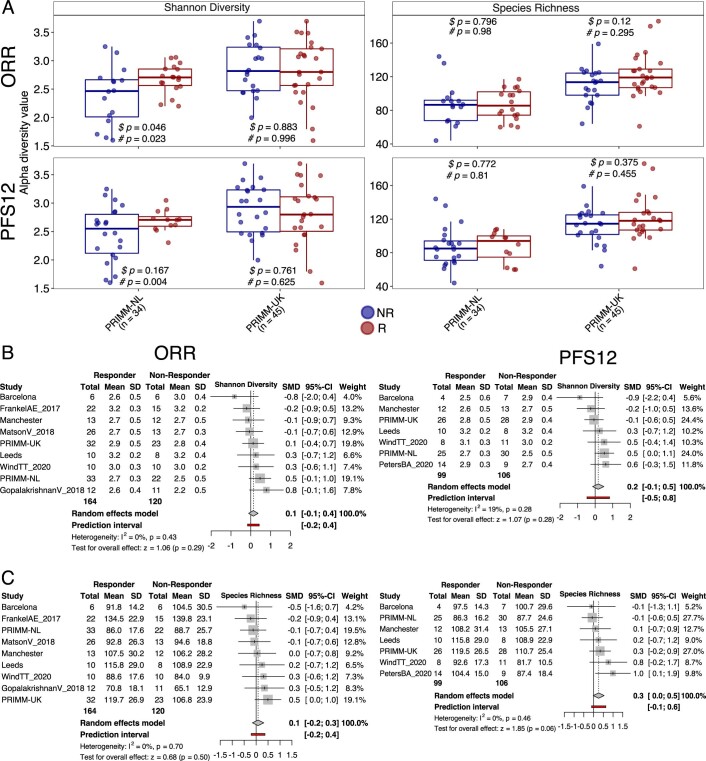
Associations between alpha diversity and response. **(a)** Alpha diversity measures in the two PRIMM cohorts. *# p* refers to P values calculated using limma linear models including PPI, antibiotic and steroid use, gender, performance status, previous therapy, age, plant-based diet index, unhealthy plant-based diet index, mediterranean diet score and ICI in the model. *$ p* refers to p values calculated using the Wilcoxon Rank-Sum Test. The lower and upper hinges of boxplots correspond to the 25th and 75th percentiles, respectively. The midline is the median. The upper and lower whiskers extend from the hinges to the largest (or smallest) value no further than ×1.5 interquartile range from the hinge, defined as the distance between the 25th and 75th percentiles. Meta-analysis results obtained using standardized mean differences between responders and nonresponders using **(b)** Shannon diversity and **(c)** species richness. The centre of the error bars (grey shaded squares) represent the standardized mean difference for each cohort.

First, we assessed the quantitative taxonomic composition of the microbiome in relation to ORR, defined as a complete/partial response or disease stability as assessed by RECIST v1.1 6 months following initiation of ICI treatment and consistent with the response definition from previous microbiome–ICI studies^11,12,16^. Using this definition, we found a borderline statistically significant difference in the microbiome composition of responders compared to nonresponders in the PRIMM-UK cohort (permutational multivariate analysis of variance (PERMANOVA) *P* = 0.05, Fig. 1a), but not in PRIMM-NL (*P* = 0.61, Fig. 1a). Progression-free survival at 12 months (PFS12), defined as duration of a complete/partial response or disease stability as assessed by RECIST v1.1 12 months following initiation of ICI treatment, was available for all patients in PRIMM-NL and 98% of patients in PRIMM-UK and showed similar association patterns (Fig. 1a). Anthropometric factors (e.g., body mass index (BMI), age and gender), information on previous nonimmunotherapy treatments, previous drug therapies (e.g., antibiotics, proton pump inhibitors (PPIs) and steroids) and dietary patterns from food frequency questionnaires (FFQs), which we converted into dietary indices (i.e., the modified Mediterranean diet score and plant-based diet index; Supplementary Table 2) were collected in these cohorts for the majority of patients and thus considered in a multivariate analysis (Fig. 1b). Consistently with the univariate analysis, we found that in the PRIMM-UK cohort, ORR and PFS12 were the two variables explaining the largest variance (*P* = 0.09 and *P* = 0.018, respectively), whereas in PRIMM-NL, we found the use of PPIs, gender, Eastern Cooperative Oncology Group performance status and previous antitumor therapy to significantly explain the variance (PERMANOVA *P* < 0.05) potentially hiding any PFS12- and ORR-associated signatures. Alpha diversity was generally not associated with response to ICIs, with only ORR in PRIMM-NL reaching statistically higher Shannon diversity in responders, but not richness, after accounting for confounding factors (Extended Data Fig. 1). We thus noticed cohort-dependent variability in the association between microbiome composition and population characteristics, including differences in dietary patterns (Supplementary Fig. 1), that could in part explain the difference in the link between the microbiome and ORR/PFS12 in the two PRIMM cohorts.

**Extended Data Fig. 2:**
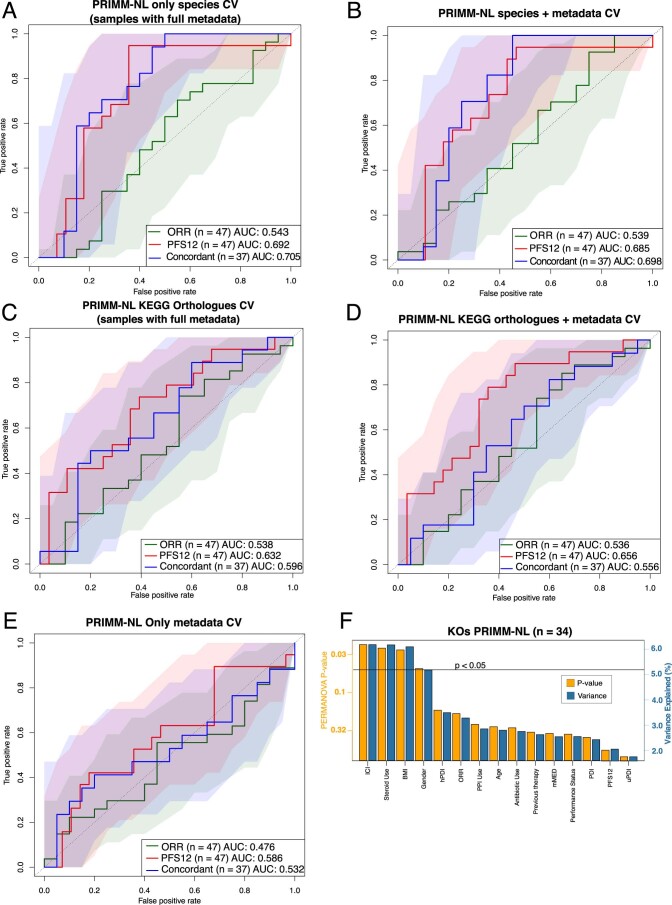
Machine learning association analysis of the microbiome and response in PRIMM-NL. (**a-e**) Machine learning association analysis of the microbiome and response using either metadata alone or in combination with taxonomic (species abundance) and functional profiles (KEGG orthologs’ abundances) in PRIMM-NL. AUC-ROC curves are computed using LASSO models trained using 100-repeated fivefold-stratified cross-validations. Metadata used in the models included: age, gender, performance status, PPI use, antibiotic use, steroid use, ICI and previous therapy. Shaded areas represent AUC-ROCs from each individual machine learning model. (**f**) Multivariate analysis showing the amount of inferred variance explained (R2, blue vertical bars) by each identified covariate and their respective p value (orange vertical bars) as determined by PERMANOVA on KEGG clr-transformed relative abundances.

**Extended Data Fig. 3:**
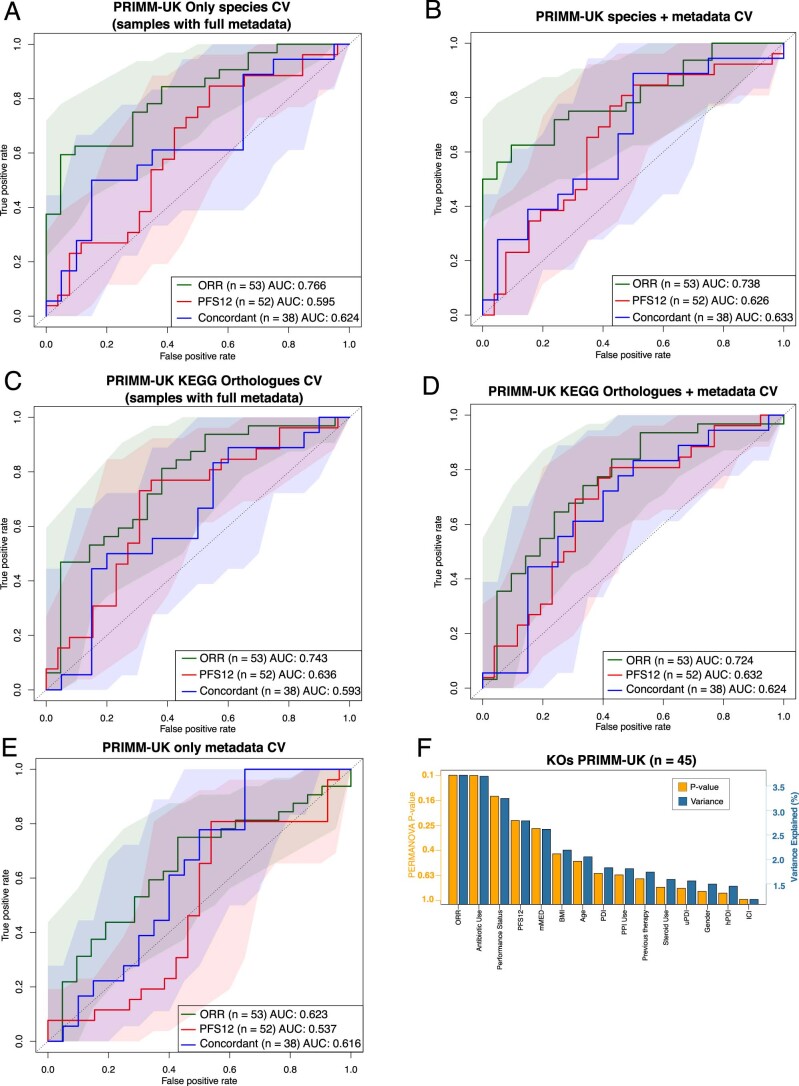
Machine learning association analysis of the microbiome and response in PRIMM-UK. (**a-e**) Machine learning association analysis of the microbiome and response using either metadata alone or in combination with taxonomic (species abundance) and functional profiles (KEGG orthologs’ abundances) in PRIMM-UK. AUC-ROC curves are computed using LASSO models trained using 100-repeated fivefold stratified cross-validations. Metadata used in the models included: age, gender, performance status, PPI use, antibiotic use, steroid use, ICI and previous therapy. Shaded areas represent AUC-ROCs from each individual machine learning model. (**f**) Multivariate analysis showing the amount of inferred variance explained (R2, blue vertical bars) by each identified covariate and their respective p value (orange vertical bars) as determined by PERMANOVA on KEGG clr-transformed relative abundances.

Response-associated microbiome features may not be reflected at the whole-microbiome level by common beta diversity summary metrics. We thus used a Lasso-based machine learning framework^14,17^ to estimate the prediction ability of the combination of taxonomic and functional features of the microbiome to segregate responders and nonresponders (cross-validation setting with nested cross-validation for feature selection; Methods and Fig. 1c). When exploring the relative abundance of each detected microbial species in this framework, we found substantial microbiome prediction capability in PRIMM-NL when using PFS12 as the endpoint (area under the receiver operating characteristic curve (AUC-ROC) 0.64) and in PRIMM-UK when using ORR (AUC-ROC 0.78), but ORR in PRIMM-NL and PFS12 in PRIMM-UK achieved much lower prediction levels (0.53 and 0.57, respectively). We then looked at the predicted functional potential of the microbiome by estimating the presence and relative abundance of microbial gene families (specifically Kyoto Encyclopedia of Genes and Genomes (KEGG) ortholog families; Fig. 1c). Here, the AUC-ROC exceeded 0.59 for both datasets and endpoints (ORR and PFS12). When we only considered patients who did not progress between 6 and 12 months (Fig. 1c), the prediction capabilities were more consistent across cohorts (0.62 for PRIMM-NL and 0.71 for PRIMM-UK when using species; 0.68 for PRIMM-NL and 0.72 for PRIMM-UK when using gene families). Clinical metadata fields were not predictive for response when considered alone and did not provide any clear improvements over microbiome features (Extended Data Figs. 2 and 3), suggesting these associations are not substantially influenced by potential confounding factors. These findings show that the gut microbiome does have a relevant association with response to ICIs but appears to be cohort dependent and is likely due to features of the microbial community that are not responsible for large shifts in the global microbiome composition.

### Limited reproducibility of response predictions across cohorts

**Fig. 2:**
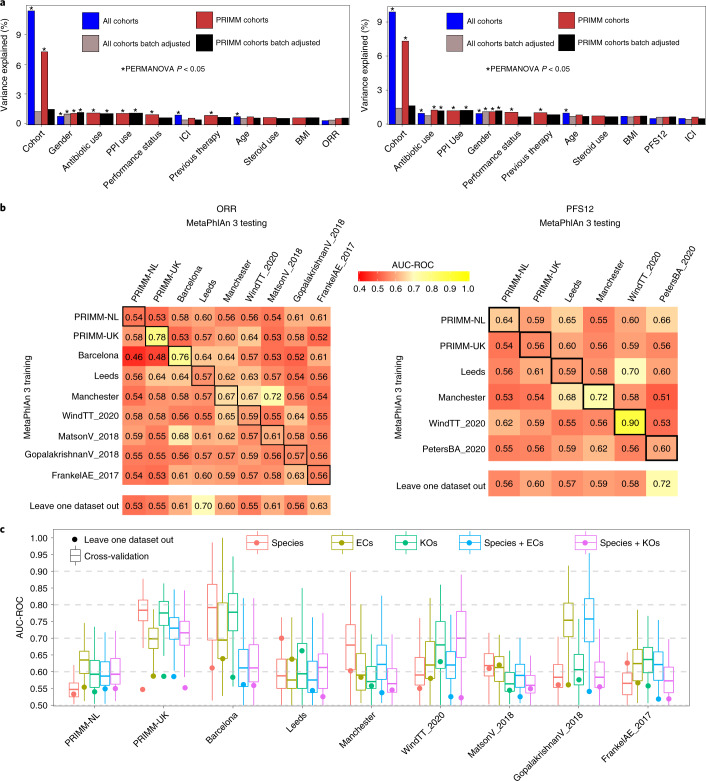
Integrated analysis of newly sequenced and publicly available datasets for cross-cohort response–microbiome association. **a**, Contribution of variables to the overall microbial community composition highlights the heterogeneity of the microbiome structure across cohorts that has a substantially higher effect than both anthropometric and clinical parameters. We either used all available cohorts or newly sequenced cohorts for which additional metadata were available. Batch-correction methods were applied to species-level abundances prior to distance calculations. The plot on the left uses ORR as the outcome variable, whereas the plot on the right adopts PFS12. **b**, Prediction matrix for microbiome-based prediction of response assessed via ORR (left matrix) and PFS12 (right matrix) within each cohort (values on the diagonal), across pairs of cohorts (one cohort used to train the model and the other for testing) and in the leave-one-cohort-out setting (training the model on all but one cohort and testing on the left-out cohort). We report the AUC-ROC values obtained from Lasso models on species-level relative abundances. Values on the diagonal refer to the median AUC-ROC values of 100-repeated fivefold-stratified cross-validations. Off-diagonal values refer to AUC-ROC values obtained by training the classifier on the cohort of the corresponding row and applying it to the cohort of the corresponding column. The leave-one-out row refers to the performances obtained by training the model using all but the cohort of the corresponding column and applying it to the cohort of the corresponding column. The same prediction matrix using functional microbiome profiles are available in Extended Data Fig. 4. **c**, ORR (*n* = 284) cross-validation AUC-ROC values obtained from Lasso models trained using 100-repeated fivefold-stratified cross-validations (boxplots) and leave-one-dataset-out AUC-ROC values from Lasso models obtained by training the model using species-level relative abundances and all but the corresponding (circles). The lower and upper hinges of boxplots correspond to the 25th and 75th percentiles, respectively. The midline is the median. The upper and lower whiskers extend from the hinges to the largest (or smallest) value no further than 1.5× interquartile range from the hinge, defined as the distance between the 25th and 75th percentiles. EC, enzyme category.

We next performed an integrated and cross-cohort analysis of the PRIMM cohorts together with three additional smaller cohorts from Barcelona, Leeds and Manchester, as well as five publicly available cohorts with consistent metagenomic and immunotherapy response data. The three cohorts we provide here in addition to PRIMM-UK and PRIMM-NL consist of a total of 55 samples (Table 1) and include patients who also collected stool samples and have metadata available as for the PRIMM cohorts. In this analysis, we also included four previously published cohorts with available metagenomic sequencing data and with ORR endpoints available^5,6,12,16^ and two with PFS12 endpoints available^16,18^. As expected, the different datasets exhibited strong cohort-dependent effects in the microbial population, with ‘cohort’ explaining nearly ten times more variance than any other variable (Fig. 2a). Restricting the analysis to the datasets we sequenced, the batch effects were less strong, but even when batch-correction adjustment using an empirical Bayes framework^19^ on centered log-ratio-transformed species-level relative abundances was applied, ORR and PFS12 still only explained a limited fraction of the total variation in the microbiome (Fig. 2a) and were not associated with increased or decreased alpha diversity (Extended Data Fig. 1b,c).

**Extended Data Fig. 4:**
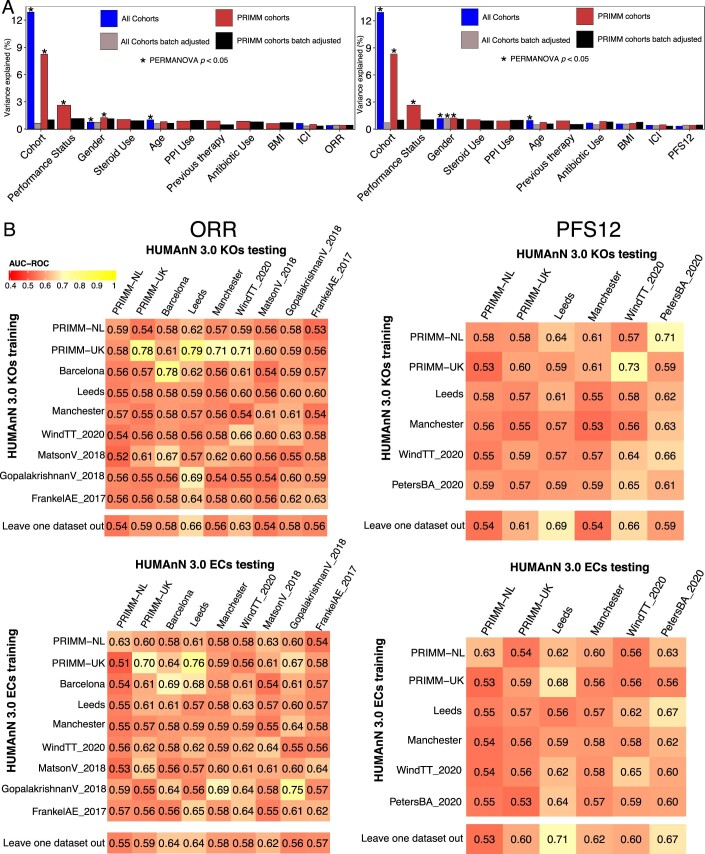
Cross-cohort response–microbiome associations at the functional level. **(a)** Contribution of variables to the overall microbial community composition. Batch-correction methods were applied to KEGG abundances prior to distance calculations. The plot on the left uses ORR as the outcome variable, whereas the plot on the right adopts PFS12. (**b**) Prediction matrix for microbiome-based prediction of response assessed via ORR (left matrix) and PFS12 (right matrix) within each single cohort (values on the diagonal), across pairs of cohorts (one cohort used to train the model and the other for testing), and in leave-one-cohort-out setting (training the model on all but one cohort and testing on the left-out cohort). We report the AUC-ROC values obtained from LASSO models on KEGG relative abundances (top) and level 4 enzyme categories (bottom). Values on the diagonal refer to the median AUC-ROC values of 100-repeated fivefold stratified cross-validations. Off-diagonal values refer to AUC-ROC values obtained by training the classifier on the cohort of the corresponding row and applying it to the cohort of the corresponding column. The leave-one-out row refers to the performances obtained by training the model using all but the cohort of the corresponding column and applying it to the cohort of the corresponding column.

**Extended Data Fig. 5:**
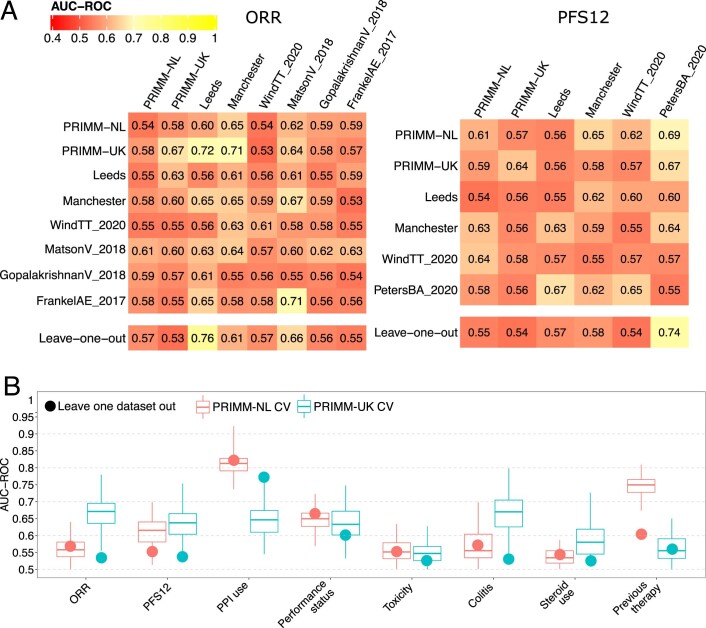
Machine learning association analysis using random forest. (**a**) Prediction matrix for microbiome-based prediction of response assessed via ORR (left matrix) and PFS12 (right matrix) within each single cohort (values on the diagonal), across pairs of cohorts (one cohort used to train the model and the other for testing), and in leave-one-cohort-out setting (training the model on all but one cohort and testing on the left-out cohort). We report the AUC-ROC values obtained from Random Forest models on species-level relative abundances. Values on the diagonal refer to the median AUC-ROC values of 100-repeated fivefold stratified cross-validations. Off-diagonal values refer to AUC-ROC values obtained by training the classifier on the cohort of the corresponding row and applying it to the cohort of the corresponding column. The leave-one-out row refers to the performances obtained by training the model using all but the cohort of the corresponding column and applying it to the cohort of the corresponding column. (**b**) Cross-validation AUC-ROC values obtained from Random Forest models trained using 100-repeated fivefold stratified cross-validations (boxplots) and leave-one-dataset-out AUC-ROC values from Random Forest models obtained by training the model using species-level relative abundances and all but the corresponding PRIMM cohort (circles). PRIMM-NL (n = 55) and PRIMM-UK (n = 55). The lower and upper hinges of boxplots correspond to the 25th and 75th percentiles, respectively. The midline is the median. The upper and lower whiskers extend from the hinges to the largest (or smallest) value no further than ×1.5 interquartile range from the hinge, defined as the distance between the 25th and 75th percentiles.

Next, we assessed whether and how a microbiome-based machine learning model can predict ICI response in samples or whole cohorts not considered in the training of the classification model^13,14,20^ (Fig. 2b). We confirmed some single datasets had ORR-prediction levels higher than PRIMM-UK (e.g., Barcelona AUC-ROC 0.76) and PFS12-prediction levels higher than PRIMM-NL (WindTT_2020 AUC-ROC 0.90), but the higher AUC-ROC values usually corresponded to small cohorts on which the estimations are more variable when assessing cross-validation, and overall, the AUC-ROC values for the additional cohorts only occasionally exceeded 0.6 (Fig. 2b). The same sample-size limitations may also explain the AUC-ROC values obtained when a response-specific microbiome model was fitted on one cohort and then tested on a different one; such values reached 0.7 in only two cases and exceeded 0.6 in around one-third of the cases (31.4%), with large variability among predictions. Interestingly, more consistent results, albeit still limited in their absolute values, were obtained when the model was fit on all but one cohort and applied on the left-out one (Fig. 2b). In this setting, which tried to alleviate cohort-specific effects by considering all but one cohort in the same model, we produced prediction values averaging 0.59 and 0.60 across the ORR and PFS12 endpoints, respectively, encompassing largely overlapping datasets. Functional characteristics of the microbiome profiled via classifications such as the enzyme category system^21^ or KEGG orthology (KO)^22^ achieved higher predictions of response compared to species relative abundances in several cases (Fig. 2c and Extended Data Fig. 4) but with higher variability and thus less cross-cohort consistency. These taxonomic and functional predictions (even when combined; Fig. 2c) were not dependent on the specific machine learning approach, as adopting random forest instead of Lasso produced similar results (Extended Data Fig. 5), and overall, they do not point to substantial cross-cohort reproducible links between the microbiome and ICI response. Nevertheless, they still highlight that profiling the microbiome at the species and gene-family levels in consistent cohorts can provide relevant indications of tumor response to ICIs.

### Few reproducible biomarkers of response across cohorts

**Fig. 3:**
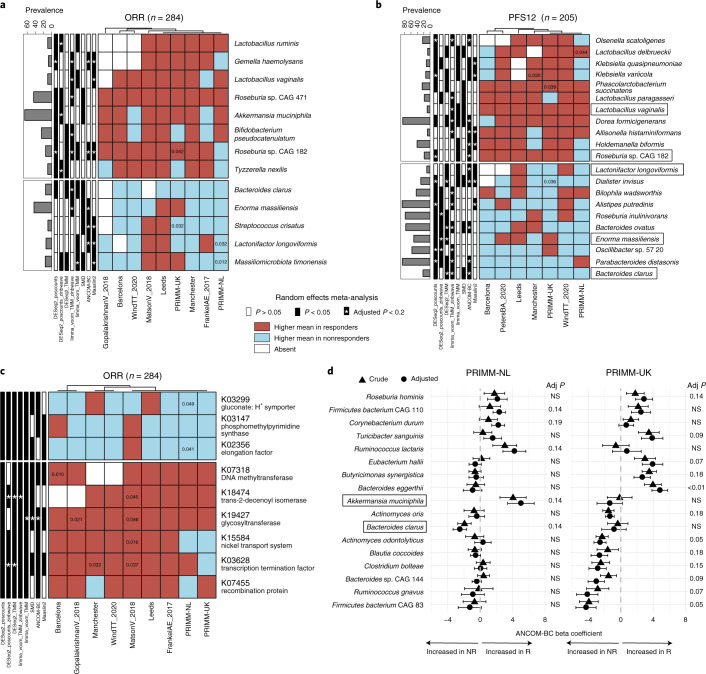
A panel of potential taxonomic and function microbiome biomarkers for response across cohorts. **a**, Species associated with ORR identified by a meta-analysis using different differential abundance methods. Species shown have random-effects model *P* values < 0.05 in at least three methods. Values inside the cells refer to unadjusted *P* values < 0.05 obtained by two-tailed Wilcoxon tests on differences in the relative abundance of responders and nonresponders. The color of the cell was determined by comparing the mean relative abundance in responders to nonresponders; if the mean was higher in responders, then the cells were colored red; if it was higher in nonresponders, then it was colored blue. **b**, Species associated with PFS12 identified by a meta-analysis using different differential abundance methods. Species shown have random-effects model *P* values < 0.05 in at least three methods. Values inside the cells refer to unadjusted *P* values < 0.05 obtained by two-tailed Wilcoxon tests on differences in the relative abundance of responders and nonresponders. **c**, KOs associated with response status identified by a meta-analysis using different differential abundance methods. The KEGG orthologues shown have random-effects model *P* values < 0.05 in at least six methods. Values inside the cells refer to unadjusted *P* values < 0.05 obtained by two-tailed Wilcoxon tests on differences in the relative abundance of responders and nonresponders. **d**, Species associated with ORR in the two PRIMM cohorts (PRIMM-NL (*n* = 47) and PRIMM-UK (*n* = 53)) before and after adjusting for covariates that included PPI, antibiotic and steroid use; gender, performance status; previous therapy; age; and ICIs. Species shown have covariate-adjusted multiple hypothesis testing-corrected *q* < 0.2 in one of the cohorts identified by ANCOM-BC. Symbols (circles and triangles) show the ANCOM-BC beta coefficient, and error bars represent standard error. Adj, adjusted; NR, nonresponders; R, responders; SMD, standardized mean differences.

**Extended Data Fig. 6:**
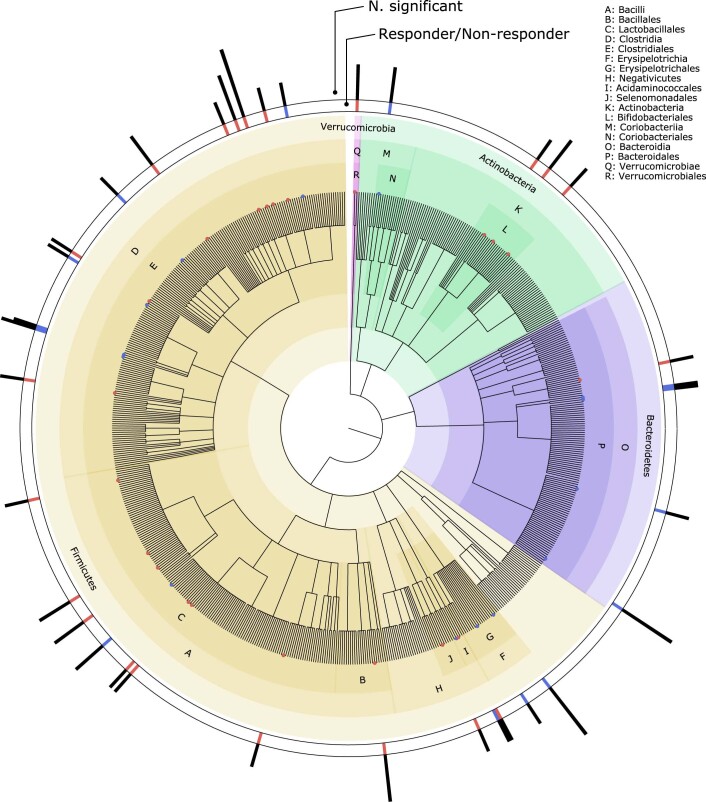
Taxonomic overview of species associations with response. Cladogram showing species associated with Responders (red) and nonresponders (blue) using ORR and identified by a minimum of 2 meta-analysis methods. The height of the outer bar plots reflects the number of meta-analysis methods supporting the association.

**Extended Data Fig. 7:**
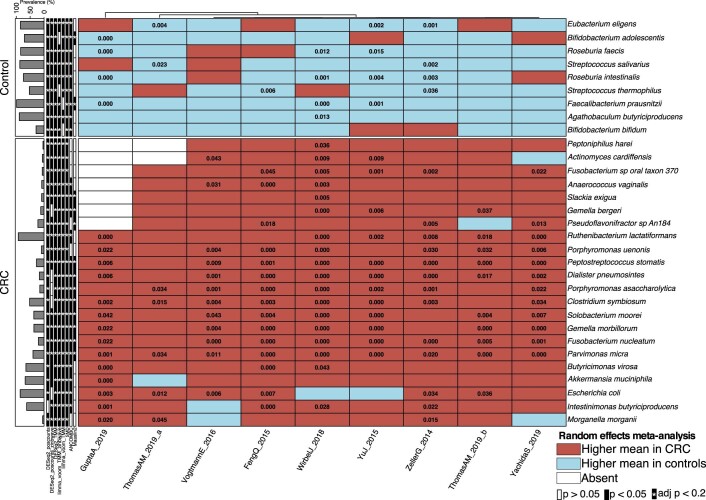
Reproducible biomarkers for colorectal cancer across cohorts. Species associated with CRC identified by a meta-analysis using different differential abundance methods. Species shown have random-effects model p values < 0.05 in at least 6 methods out of 8 methods. Values inside the cells refer to unadjusted p values < 0.05 obtained by two-tailed Wilcoxon tests on differences in the relative abundance of patients with CRC and controls.

We then looked for microbial taxa or functions consistently associated with response to ICIs across the available cohorts in relation to ORR and PFS12 (Fig. 3, Extended Data Fig. 6 and Supplementary Tables 3 and 4). This analysis revealed two uncultivated *Roseburia* species associated with response; *Roseburia* sp. CAG:182 (also associated with response when using PFS12) and *Roseburia* sp. CAG:471 increased on average in responders consistently across all datasets with ORR endpoints available. Of note, *Roseburia* sp. CAG:182 was found to be in the panel of the five bacterial species most associated with favorable nutritional and cardiometabolic health markers in a recent large metagenomic population-based study^20^, with a particularly strong inverse correlation with the inflammatory surrogate glycoprotein acetyl^23^. With respect to those patients for whom PFS12 data were available, *Phascolarctobacterium succinatutens* and *Lactobacillus vaginalis* were both enriched in responders across all seven datasets, and each was detected as significant by three of the eight meta-analysis approaches using the same significance thresholds. *A. muciniphila* and *Dorea formicigenerans* were two species with high overall prevalence (65.8% and 85.9%, respectively) associated with ORR and PFS12 (by three and six meta-analysis methods, respectively). *Bacteroides clarus* instead exhibited higher relative abundances in nonresponders, both for ORR and PFS12, for all the seven datasets in which it can be detected and was significantly associated with nonresponders in PRIMM-NL after covariate adjustment (Fig. 3d). No single bacterium was a fully consistent biomarker of response across all datasets; however, applying the same meta-analysis methods in the context of colorectal cancer as a methodological control confirmed strong and consistent biomarkers across cohorts, reinforcing previous meta-analyses^13,14^ (Extended Data Fig. 7 and Supplementary Table 5). We then focused on the panel of species associated with responders by at least three meta-analysis methods, which showed a high concordance with results from a Bayesian framework for compositional sequencing data (Pibble models; Supplementary Fig. 2). This panel contains taxa generally associated with healthy host conditions, including species with probiotic potential (*Bifidobacterium pseudocatenulatum*, *Lactobacillus ruminis* and *Turicibacter sanguinis*), as well as species involved in butyrate production (*Roseburia* spp., *Eubacterium hallii* and *Butyricimonas synergistica*). *A. muciniphila* also belonged to this group, with significant association also in the PRIMM-NL study, supporting previous findings on its role in immunotherapy^11^, whereas the presence of *Ruminococcus gnavus* as a biomarker of nonresponders in PRIMM-UK is in line with multiple reports of its association with poor cardiometabolic health^20^ and several diseases^24–26^. Expanding our analysis to gut microbiome members beyond bacteria and archaea, we found no association between response and the presence of *Blastocystis* (8.9% of individuals were *Blastocystis* positive, the highest prevalence of eukaryotic organisms found; Supplementary Table 6) or viruses (Supplementary Fig. 3).

**Extended Data Fig. 8:**
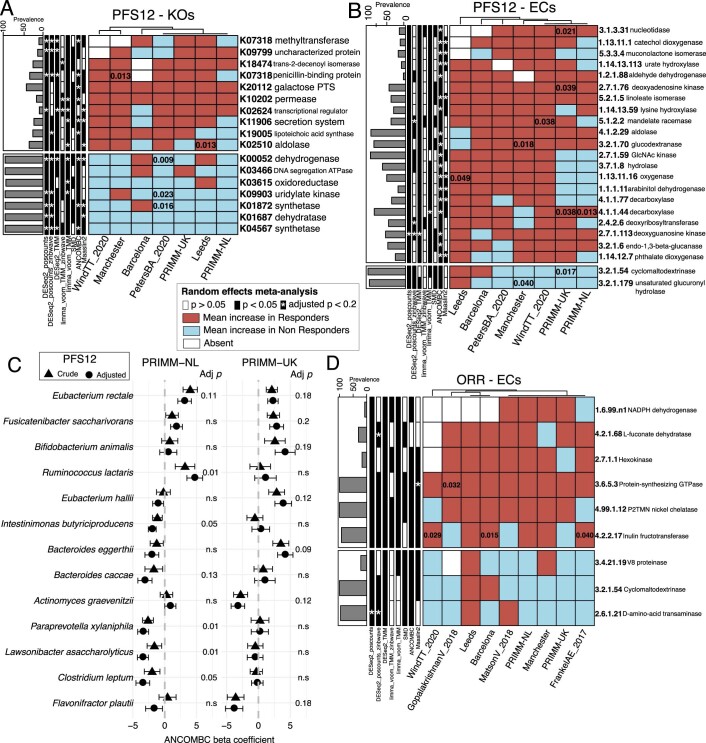
Microbiome biomarkers of response across cohorts. (**a**) KEGG orthologues associated with PFS12 identified by a meta-analysis using different differential abundance methods. KEGGs shown have random-effects model p values < 0.05 in at least 6 methods out of 8 methods. Values inside the cells refer to unadjusted p values < 0.05 obtained by two-tailed Wilcoxon tests on differences in the relative abundance of responders and nonresponders. (**b**) Level 4 enzyme categories associated with PFS12 identified by a meta-analysis using different differential abundance methods. ECs shown have random-effects model p values < 0.05 in at least 6 methods out of 8 methods. Values inside the cells refer to unadjusted p values < 0.05 obtained by two-tailed Wilcoxon tests on differences in the relative abundance of responders and nonresponders. (**c**) Species associated with PFS12 in the two PRIMM cohorts before and after adjusting for confounders that included PPI, antibiotic and steroid use, gender, performance status, previous therapy, age and ICI. PRIMM-NL (n = 47) and PRIMM-UK (n = 52). Species shown have covariate-adjusted multiple hypothesis testing-corrected q < 0.2 in one of the cohorts identified by ANCOM-BC. Symbols (circles and triangles) show the ANCOM-BC beta coefficient and error lines represent the standard error. (**d**) Level 4 enzyme categories associated with ORR identified by a meta-analysis using different differential abundance methods. ECs shown have random-effects model p values < 0.05 in at least 6 methods out of 8 methods. Values inside the cells refer to unadjusted p values < 0.05 obtained by two-tailed Wilcoxon tests on differences in the relative abundance of responders and nonresponders.

When assessing treatment response with the predicted functional potential of the gut microbiome, we identified a number of KOs increased in responders (Supplementary Tables 7 and 8). These included a glycosyltransferase (*epsJ*) known to be essential for pellicle formation^27^, with abundance contributions from *Intestinimonas butyriciproducens, Bifidobacterium angulatum* and *Bifidobacterium pseudoangulatum* and significant in six of the eight meta-analysis approaches used (Fig. 3c). We also detected an increased abundance of DNA adenine methylases, which are part of restriction-modification systems and can influence the expression of virulence genes^28^, in responders using both ORR (Fig. 3c) and PFS12 (Extended Data Fig. 8), with contributions mostly from *I. butyriciproducens*. We also found a gluconate symporter to be increased in nonresponders, with abundance contributions mostly from *Rumminococcus torques*, *Escherichia coli* and *Klebsiella pneumoniae*. Gluconate is an important part of mucin 2 and is involved in binding of flagella^29^.

### Clinical parameters linked to the microbiome

**Fig. 4:**
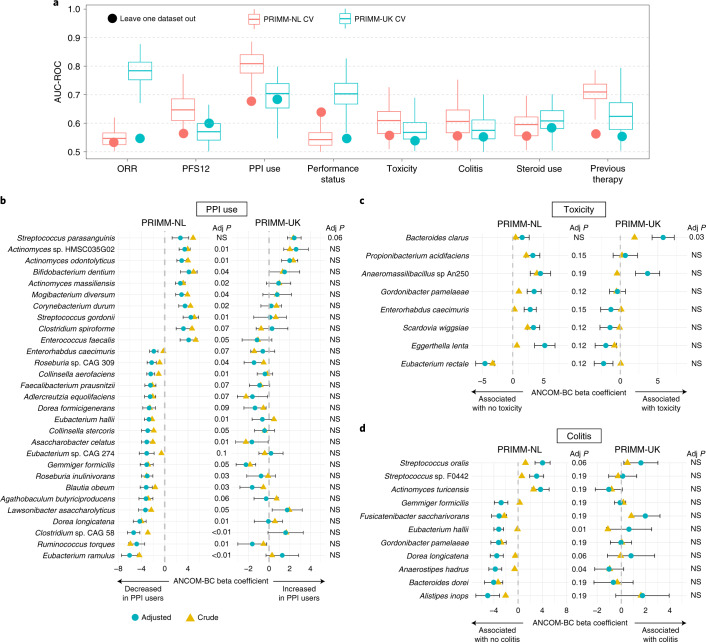
Covariate associations with the gut microbiome from the PRIMM cohorts. **a**, Cross-validation AUC-ROC values obtained from Lasso models trained using 100-repeated fivefold-stratified cross-validations (boxplots) and leave-one-dataset-out AUC-ROC values from Lasso models obtained by training the model using species-level relative abundances and all but the corresponding PRIMM cohort (circles). The lower and upper hinges of boxplots correspond to the 25th and 75th percentiles, respectively. The midline is the median. The upper and lower whiskers extend from the hinges to the largest (or smallest) value no further than 1.5× interquartile range from the hinge, defined as the distance between the 25th and 75th percentiles (PRIMM-NL, *n* = 55; PRIMM-UK, *n* = 55). **b**–**d**, Species associated with PPI use (<3 months after the start of ICI), toxicity and colitis identified by ANCOM-BC with and without covariate adjustment (PRIMM-NL, *n* = 47; PRIMM-UK, *n* = 53). Covariates included in all models were ORR, performance status, previous therapy, age, ICIs (combination of ipilimumab and nivolumab or single agent), gender and antibiotic and steroid use. PPI use was also included as a covariate when analyzing colitis and toxicity. Species shown have covariate-adjusted multiple hypothesis testing-corrected *q* < 0.2 in one of the cohorts identified by ANCOM-BC. Symbols (circles and triangles) show the ANCOM-BC beta coefficient, and error bars represent standard error.

**Extended Data Fig. 9:**
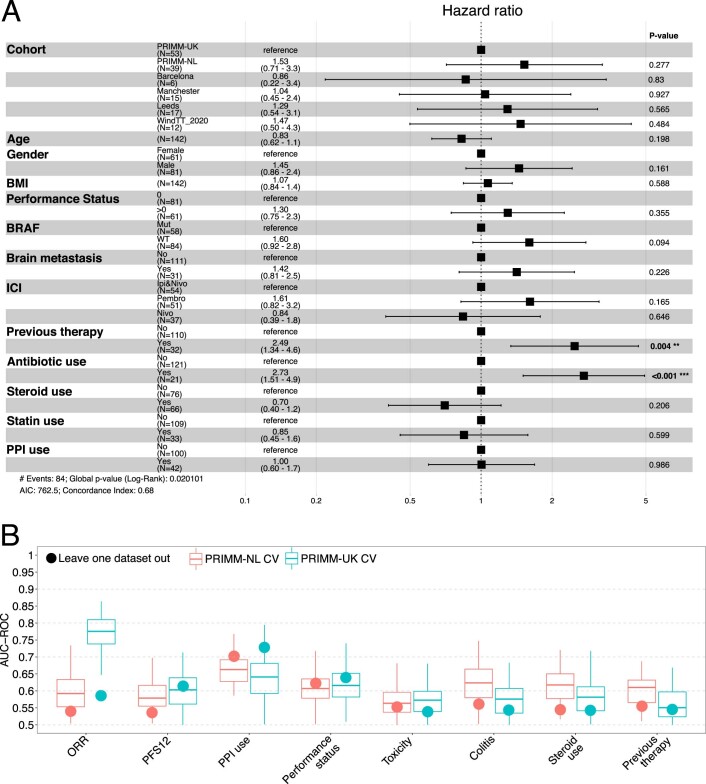
Clinical parameters associated with response and the microbiome. **(a)** Forest plot showing Cox logistic regression multivariate analysis of progression-free survival. Error lines represent the 95% confidence interval of the hazard ratio. **(b)** Cross-validation AUC-ROC values obtained from LASSO models trained using 100-repeated fivefold stratified cross-validations (boxplots) and leave-one-dataset-out AUC-ROC values from LASSO models obtained by training the model using KEGG relative abundances and all but the corresponding PRIMM cohort (circles). PRIMM-NL (n = 55) and PRIMM-UK (n = 55). The lower and upper hinges of boxplots correspond to the 25th and 75th percentiles, respectively. The midline is the median. The upper and lower whiskers extend from the hinges to the largest (or smallest) value no further than ×1.5 interquartile range from the hinge, defined as the distance between the 25th and 75th percentiles.

Patients with unresectable advanced melanoma have potential clinical confounding factors that can affect both the microbiome and might obfuscate the association between the pre-ICI microbiome composition and clinical response. A consistent panel of clinical information within our cohorts, ranging from PPI usage before treatment to performance status and toxicity, allowed us to test whether and how such parameters were connected with microbiome composition and the microbiome signatures of response to ICIs. In line with previous reports^30–32^, we found that PPIs had the strongest link with the microbiome in both PRIMM-NL and PRIMM-UK (Fig. 4a); however, PPI use was not associated with response or PFS12 (Extended Data Fig. 9) and so is not per se a factor biasing the microbiome–response associations. Moreover, ORR in PRIMM-UK reached the same level of prediction based on the taxonomic-based microbiome model, which confirmed that the response–microbiome signature is independent of that of PPI use given the limited overlap in nonresponding patients who reported PPI use before ICI treatment (*P* = 1, Fisher’s exact test). When looking at the functional potential, we also found that PPI use was the covariate best predicted by the gut microbiome in a leave-one-dataset-out setting for both PRIMM cohorts (Extended Data Fig. 9).

Performance status was also quite well inferred from the microbiome in PRIMM-UK and also in PRIMM-NL when considering the leave-one-dataset-out strategy (Fig. 4a and Extended Data Fig. 9). ICI-induced toxicity and colitis, for which preliminary immunological, genomic and microbiology biomarkers are available^33^, were less associated with the microbiome in our study, in which 43% and 36% of individuals experienced grade 3 or higher immune-related adverse events in PRIMM-UK and PRIMM-NL, respectively (Methods). Within PRIMM-NL, *E. hallii* and *Anaerostipes hadrus* were both found to be significantly associated with the absence of colitis before ICI treatment, whereas no significant species emerged from the analyses of PRIMM-UK samples. *Bacteroides clarus* was significantly associated with the development of ICI-induced toxicity within PRIMM-UK and was also associated with nonresponse in PRIMM-NL and the meta-analysis. *Eubacterium rectale* was significantly associated with the absence of toxicity in PRIMM-NL (and nonsignificantly in PRIMM-UK) and was associated with response when using PFS12 (Extended Data Fig. 8) in both PRIMM-UK and PRIMM-NL. Overall, several clinical parameters were found to be linked with the composition of the gut microbiome, but such associations appeared to be rather independent of ICI response.

## Discussion

In this study, we present the largest metagenomic study to date to identify gut microbiome associations with ICI response by integrating published melanoma cohorts (*n* = 147) with five new cohorts (*n* = 165). This large set of real-world cohorts was meta-analyzed extending previous attempts^34^ to verify whether the partially conflicting biomarkers of response to ICI found in single small datasets^5,11,12,16,18^ could be reconciled. Using ORR and PFS12 by RECIST v1.1 at 6 and 12 months, respectively, as endpoints, we confirmed both the presence of cohort-specific biomarkers and the absence of taxonomic or functional microbiome biomarkers that are consistent across all datasets. The lack of solid cross-study reproducible microbial biomarkers of ICI response is not attributable to analytical choices, as applying the same meta-analysis methods in the context of colorectal cancer confirmed the strong and consistent biomarkers across cohorts described elsewhere^13,14^. The variability of the microbiome link to tumor response to ICIs was confirmed by machine learning analysis, which suggested that the microbiome is predictive of the response in some, but not all, cohorts. Importantly, the task of cross-cohort prediction to identify ICI responders and nonresponders in cohorts different from those used to develop the machine learning model clarified that it is still very difficult to overcome the limitations of population and microbiome heterogeneity to predict response reliably. Although we confirmed that the microbiome holds the potential to support the clinical practice for the treatment of patients with melanoma, as shown via fecal microbiota transplantation pilots^7,8^, several limitations need to be overcome before we find robust microbial biomarkers.

There are multiple limitations of linking ICI and the gut microbiome. The rather small sample sizes of each individual cohort, despite the new additions in this work, are certainly one aspect, but it is inherently difficult to collect very large, properly annotated sample sizes in this clinical setting. Our work also highlighted previously overlooked limitations in defining response (as seen by differences in microbiome links to ORR and PFS12 in the same cohorts) and the vast number of potentially confounding factors for which it is difficult to adjust for. Moreover, the generally strong cohort microbiome effects even within multicenter studies appear to be much stronger in the analyzed melanoma ICI treatment trials than in other clinical settings. Current methodology to profile the gut microbiome can survey in-depth taxonomic and functional aspects of the microbial communities but generally lacks the ability to characterize the microbiome features at the immunological interface.

It is unclear why PFS12 was seen to have substantially higher microbiome prediction capability in PRIMM-NL, whereas ORR performed substantailly better within the PRIMM-UK cohort. Nevertheless, aside from the large sample size of the combined cohorts, our study has multiple strengths; samples for this study came from three European countries and a number of publicly available datasets, representing a diverse and heterogeneous cohort of real-world patients, and we account for a variety of important confounders and prognostic factors that are often overlooked.

The gut microbiome has an important role to play in ICI response^7,8^, as we also confirmed here, but this role appears likely to be more complex than previously reported in initial studies, extending beyond differential microbial abundances and encompassing complex interactions of the gut microbiome with clinical predictors and biological factors that may be specific to geographies in patients who benefit from treatment with ICIs. The gut microbiome is unique in each individual, even when considering identical twins^20,35^, and a large fraction of this uniqueness is encoded at the level of single strains^36–38^. Therefore, analytical methods are needed for deeper exploration of such individual-specific microbial diversity, but such resolution would also require sample sizes in the order of several thousand individuals to deal with the substantial increase in the number of relevant microbial features. Understanding the functional output of the gut microbiota, immunological relations that exist between specific microbiota and the host at the strain level or even perhaps examination of the gut mucosa as distinct from stool will allow us further insight into the mechanisms by which the gut microbiome impacts treatment with ICIs. Links between the gut microbiome and ICI response can also be partially cancer specific and influenced by many factors that are inherently difficult to account for, and it will thus be important to extend analyses and meta-analysis beyond the cutaneous subtype of melanoma to include the uveal and mucosal subtypes, as well as other cancers for which ICI treatments are available. Even within the same cancer (sub)type, it is unlikely that the same microbiome features can reflect the uniqueness of the genetic and immune characteristics of each tumor, meaning that expectations on the existence of a universal, very accurate and highly reproducible link between the human microbiome and ICI response should be lowered. Continued efforts should thus be put into performing metagenomic investigations at substantially larger scales with improved representation of distinct populations while controlling for clinical covariates and ensuring that samples are collected and processed in the same manner and using the same techniques. This study improves the current understanding of the link between the gut microbiome and ICI response and sheds light on the complexities of microbiome science in human disease.

## Methods

### The prospective PRIMM cohorts and the other enrolled cohorts

We prospectively collected fecal samples from patients receiving ICIs between August 2015 and January 2020 for patients with advanced cutaneous melanoma treated in the United Kingdom (PRIMM-UK study) and the Netherlands (PRIMM-NL study, made up of eligible patients from the COLIPI, POINTING and OncoLifeS studies). For the present study, we analyzed stool samples collected prior to treatment with ICIs (at baseline). PRIMM-UK (NCT03643289) is sponsored by East and North Hertfordshire NHS Trust with ethical approval from King’s College London. OncoLifeS (Medisch Ethische Toetsingsingscommissie (METc) 2010/109), COLIPI (METc 2012/085, NCT02600143) and POINTING (METc 2018/350, NCT04193956) have all been approved by the METc of the University Medical Center Groningen in the Netherlands. OncoLifeS information is available on the Netherlands Trial Register (https://www.trialregister.nl/trial/7839). Patient samples within the Manchester cohort were collected with written full-informed patient consent under Manchester Cancer Research Centre Biobank ethics application 07/H1003/161 + 5 (updated in 18/NW/0092) and approval for the work under Manchester Cancer Research Centre Biobank Access Committee application 13_RIMA_01. Barcelona cohort samples were subjected to the ethical committee of Hospital Clínic of Barcelona approval (registry HCB/2015/1032). Data and samples from Leeds were collected in a study named “Developing a blood test of immunity in illness: a study examining the peripheral blood transcriptome in patients with cancer, autoimmune disease, immunodeficiency or iatrogenic immune suppression” (Research Ethics Committee (REC) reference 15/NW/0933). Informed written consent was obtained for collection of samples and data, sharing anonymized data and working with collaborators whether academic or commercial.

Samples collected within the three external cohorts of Barcelona, Manchester and Leeds were subject to similar ethical approvals. Patients who fulfilled the following criteria were eligible for these analyses: (i) histologically or cytologically confirmed nonresectable advanced (stage III or IV) cutaneous melanoma, (ii) treatment with ICIs (nivolumab, pembrolizumab or ipilimumab or a combination of ipilimumab and nivolumab) at the recommended dose as a first-line ICI and (iii) 18 years of age or older. Written informed consent was obtained from all patients. High-quality fecal samples were collected from these patients before initiation of ICI treatment (*n* = 55 for the UK cohort and *n* = 55 for the Dutch cohort). Additional patients were enrolled from cohorts outside the setting a prospective clinical trial and performed in Leeds (*n* = 19), Barcelona (*n* = 12) and Manchester (*n* = 30) between March 2015 and November 2019 but from whom fecal samples were collected at time points similar to those used in our included prospective studies. Written informed consent was obtained from all patients.

### Sample and data collection

Baseline demographics, including sex, age, BMI, Eastern Cooperative Oncology Group performance status and medication use, were collected, along with tumor staging and previous anticancer therapy data. Demographic and dietary data were collected as part of a screening visit up to 14 days before ICI treatment began. All baseline antibiotic or PPI use within 3 months of commencing ICI treatment was documented. Tumor staging took place up to 1 month before the start of treatment. Routine blood hematology and biochemistry data, including white cell subsets, platelet count, lactate dehydrogenase and albumin, were recorded at baseline and with each treatment cycle. All baseline antibiotic or PPI use within 3 months of commencing ICI was documented.

Patients received oral and written instructions regarding the stool collection procedure. Patients within PRIMM-UK and PRIMM-NL were requested to collect approximately 3–5 ml plain feces using a collection kit that could be used at home and then store the sample in their freezer directly after collection. PRIMM-NL samples were transported to the hospital in a frozen, insulated cooling bag to prevent thawing. Due to the geographic disbursal of PRIMM-UK patients, samples were collected and placed in Thermo Fisher Scientific kits and sent by special post to the laboratory at King’s College London. After arrival in the hospital, the samples were directly stored at −80 °C. Plain stool samples from the Manchester cohort were either collected on site at the hospital and stored directly at −80 °C within 4–6 h of collection or collected into sample containers and sent by special post to the laboratories of CRUK Manchester Institute and stored directly at −80 °C upon arrival. Patients within the Barcelona cohort used the OMNIgene GUT collection kit (DNA Genotek). Fecal DNA was extracted from 1 to 14 days after sample collection using the PowerFecal DNA Isolation Kit (previously Mo Bio, currently Qiagen) and kept frozen until needed. Patients from Leeds also collected stool at home using the OMNIgene GUT collection kit (DNA Genotek), and samples were returned to the research nurse.

Radiological evaluation, consisting of a computed tomography (CT) scan of the thorax, abdomen and pelvis and magnetic resonance imaging of the brain, was performed at baseline (i.e., before the first dose of immunotherapy). A small number of patients had positron emission tomography scans with a CT component. Follow-up radiological evaluation was performed every 10–14 weeks as long as the patient received systemic therapy. Additional CT and/or magnetic resonance imaging scans were performed when there was suspicion of progression. If the first radiological evaluation after start of therapy was inconclusive, then a confirmatory scan was performed 4–12 weeks later.

### Definition of response to therapy

Response to ICI was classified according to RECIST v1.1 criteria. On the basis of radiographic response, patients were classified as responders (CR, PR or SD) or nonresponders (PD). Clinical endpoints were defined as overall response rate and PFS (defined as the time from the first dose of an ICI to the first event; i.e., disease progression or death from any cause). All patients had toxicity during or after ICI treatment, as documented by Common Terminology Criteria for Adverse Events v5. Toxicity was deemed present at or above Common Terminology Criteria for Adverse Events grade 1, as was the case with colitis. In order to include late responders in our analysis, patients with progressive disease (PD) on the first radiological evaluation but a response at the second radiological evaluation compared to baseline were also labeled responders. Patients with PD on the first radiological evaluation that was confirmed on the next follow-up scan, or patients with PD on the first radiological evaluation who were unable to complete a confirmation scan due to clinical progression or death, were labelled nonresponders.

### Dietary data collection

In 93 of the 110 (84.5%) prospectively recruited patients, dietary data were collected before treatment and within 14 days of commencing ICIs. Dietary intake was assessed through two different FFQs: the Dutch Healthy Diet-FFQ^39^ and the EPIC-Norfolk FFQ^40,41^. Food items were mapped to create one dataset. Four food-based scores were calculated to address relative dietary quality as confounder in the microbiome–response analysis: the modified Mediterranean diet score^42^, original plant-based diet index^43^, healthy plant-based diet index^43^ and unhealthy plant-based diet index^43^. The composition of scores by food groups in the UK and Dutch cohorts is given in Supplementary Table 2.

### DNA extraction and sequencing

DNA was isolated at King’s College London using the Max Core protocol. Samples with a high-quality DNA profile were further processed. Sequencing libraries were prepared using the Illumina Nextera DNA Flex Library Prep Kit according to the manufacturer’s protocols. Libraries were multiplexed using dual indexing and sequenced for 300-bp paired-end reads using the Illumina NovaSeq6000 platform according to the manufacturer’s protocols. We obtained a total of 1,283 Gb with an average of 53,919,210 reads per sample before quality control and preprocessing.

### The publicly available datasets considered

We download metagenomic data from four publicly available datasets (PetersBA_2020, GopalakrishnanV_2018, MatsonV_2018 and FrankelAE_2017) through the Sequence Read Archive using the accession numbers SRP197281 (ref. ^18^), ERP104610 (ref. ^6^), SRP116709 (ref. ^5^) and SRP115355 (ref. ^12^). Metagenomic data and metadata from WindTT_2020 (ref. ^16^) were provided by the authors of the study. These publicly available cohorts are shown in Supplementary Table 11. We excluded any samples taken after the start of ICI therapy, nonmetagenomic samples, nonfecal samples and samples with low sequencing depth (less than one million reads). We classified patients into responder and nonresponder groups according to RECIST 1.1 criteria; patients with complete or partial response, as well as stable disease at first evaluation, were classified as responders, whereas patients with PD were classified as nonresponders.

### Metagenome quality control and preprocessing

Shotgun metagenomic sequencing was performed at the NGS Core Facility at University of Trento. The quality of all sequenced metagenomes was controlled using the preprocessing pipeline implemented in https://github.com/SegataLab/preprocessing. This preprocessing pipeline consists of three main stages: (1) initial quality control by removing low-quality reads (quality score <Q20), fragmented short reads (<75 bp) and reads with more than two ambiguous nucleotides; (2) contaminant DNA removal using Bowtie 2 (ref. ^44^) and the sensitive local parameter, removing both the phiX174 Illumina spike-in and human-associated reads (hg19); and (3) sorting and splitting for the creation of standard forward, reverse and unpaired reads output files for each metagenome. Of 190 baseline samples collected across the five observational cohorts, 25 samples failed the metagenomic sequencing and preprocessing pipeline and resulted in metagenomes with <1 Gb pairs. The remaining 165 samples passed strict quality control and were processed into taxonomic and predicted pathway abundances.

### Microbiome taxonomic and functional potential profiling

The metagenomic analysis was performed following the general guidelines^45^ and relying on the bioBakery 3 environment^15,46^. bioBakery 3 is a set of integrated and improved methods for taxonomic (MetaPhlAn 3.0), strain-level (StrainPhlAn 3.0 and PanPhlAn 3.0), functional (HUMAnN 3.0) and phylogenetic (PhyloPhlAn 3.0) profiling of metagenomes. bioBakery 3 leverages a set of 99,200 high-quality and fully annotated reference microbial genomes spanning 16,800 species and the 87.3 million UniRef90 functional annotations available in UniProt as of January 2019. The taxonomic profiling and quantification of organisms’ relative abundances of all metagenomic samples were quantified using MetaPhlAn 3.0 (ref. ^47^) with default parameters. Functional potential analysis of the metagenomic samples was performed using HUMAnN 3.0 (ref. ^48^) with default parameters. In total, we identified 608 species, 510 pathways, 6,131 distinct KOs and 2,558 enzyme categories in 165 samples from the five different cohorts. Detection of *Blastocystis* in gut metagenomes was performed as previously described^49^. For viral profiling, we used 699 bacteriophages from Viral RefSeq^50^ that were found in more than 20 samples within the unbinned fraction of the metagenomic assembled genomes described by Pasolli et al.^37^ and from a set of 255 highly enriched viromes selected with the ViromeQC tool^51^. Sequences were clustered at 70% identity and further joined into ‘viral groups’ if they shared at least 90% similarity across clusters. In total, 128 groups of known viral bacteriophages were constructed. To calculate the depth of coverage of each viral group, we first mapped the raw reads of each sample against a nonredundant collection of representative sequences for each viral group. Then, a second mapping was performed on the best-matching sequence for each group and each sample. Depth of coverage was calculated with CMSeq (https://github.com/SegataLab/cmseq).

### Statistical analysis

Multivariate survival analyses were performed using Cox regression models to determine hazard ratios and 95% confidence intervals for PFS and adjusting for other clinicopathologic features using the coxph function from the R survival package (v3.2-7). We conducted PERMANOVA using the function adonis from the vegan R package (v2.5-7) with the Atchinson distance on centered log ratios using both species and KO relative abundances. The *P* and *R*^2^ values were determined by 10,000 permutations using all variables in the model. Batch correction was applied to centered log ratios using the combat function available in the Surrogate Variable Analysis (v3.38) R package. Univariate analysis was performed using both two-tailed Wilcoxon tests comparing differences in relative abundances (values inside the heatmap cells in Fig. 3a–c) and ANCOM-BC (v.1.0.1)^52^ with default parameters, a library size cutoff of 1,000 reads and no structural zero detection. Multivariate analysis was performed using ANCOM-BC, including the covariates of interest in the model formula. *P* values were corrected for multiple hypothesis testing using the Benjamin–Hochberg procedure, and a false discovery rate < 0.2 was defined as the significance threshold. In addition, we also implemented a Bayesian multinomial logistic-normal linear regression model called Pibble from the R package fido^53^, which allows for linking covariates to compositional overdispersed count data. We transformed the taxonomic relative abundances into count values for Pibble via logistic-normal distribution modeling^54^. In Pibble, the regression coefficients are ranked to determine which microbial features change the most between conditions and are identical to the rankings of absolute differentials^55^.

#### Meta-analysis

An ensemble of differential abundance methods and normalizations (eight in total) were used to estimate fold changes with their respective confidence intervals between responders and nonresponders and supplied to a random-effects model via the rem_mv function in the MetaVolcanoR R package (v.1.4.0) using the restricted maximum-likelihood estimator model. Random-effects *P* values obtained from each of these methods were corrected for multiple hypothesis testing using the Benjamin–Hochberg procedure. For methods requiring count data (i–v and vii), absolute raw counts were estimated from species-level MetaPhlAn 3 relative abundances by multiplying these values by the total number of reads for each sample. The following methods were based on an assessment of statistical methods to detect differentially abundant features in microbiome data^56^ and are available at https://github.com/mcalgaro93/sc2meta:

i. DESeq2 (v.1.30.0)^57^ with the poscounts estimator (DESeq2_poscounts);
ii. DESeq2 with the poscounts estimator and a zero-inflated negative binomial model (DESeq2_poscounts_zb), and observational weights were computed using the zinbwave package (v.1.12.0)^58^ and supplied to the DESeqDataSet class object to account for zero inflation;
iii. DESeq2 with trimmed mean of *M* values (TMM; DESeq2_TMM), where normalization factors were calculated using TMM normalization, and a negative binomial generalized log-linear model was fit to the read counts of each feature using the glmFit function and the edgeR (v.3.32.0)^59^ package;
iv. limma (v3.46.0)^60^ with TMM values (limma_voom_TMM) (the limma package includes a voom function that transforms previously normalized counts to log counts per million, estimates a mean–variance relationship and uses this to compute appropriate observational-level weights); and
v. limma with TMM values and a zero-inflated negative binomial model (limma_voom_TMM_zb) (to adapt the limma-voom framework to zero inflation, zinbwave weights were multiplied by voom weights);
  Other methods not originally considered in this assessment were also included in the meta-analysis:
vi. standardized mean differences (relative abundances were arcsine-square root transformed and followed the same procedure as in Thomas et al.^14^);
vii. ANCOM-BC (v.1.0.1)^52^, which uses a linear regression framework in log scale and accounts for sampling fraction by introducing a sample-specific bias correction that is estimated from the observed data (we used the same parameters as described in the univariate/multivariate analysis); and
viii. Maaslin2 (v.1.4.0)^61^, where logit-transformed relative abundances were normalized with total-sum scaling and supplied to the maaslin2 function using the variable of interest as a fixed effect.

### Machine learning analysis

Data preprocessing, model building and model evaluation were performed using the SIAMCAT^62^ R package (v.1.6.0). Species relative abundances were filtered to remove markers with low overall abundance (1 × 10^−4^ maximum abundance cutoff), log_10_-transformed (after adding a pseudocount of 1 × 10−^5^ to avoid nonfinite values) and standardized as *z*-scores. Functional profiles, such as KEGG orthologous and level 4 enzyme category abundance profiles were preprocessed similarly but using 1 × 10^−6^ as the maximum abundance cutoff and 1 × 10−^9^ as a pseudocount during log transformation.

#### Cross-validation

A nested cross-validation procedure was applied to calculate within-cohort accuracy (cells on the diagonal in Fig. 2b) by splitting data into training and test sets for 100-times repeated, fivefold-stratified cross-validation (balancing class proportions across folds). For each split, an L1-regularized (Lasso) logistic regression model was trained on the training set, which was then used to predict the test set. The lambda parameter was selected for each model to maximize the AUC-ROC under the constraint that the model contained at least five nonzero coefficients.

#### Cross-study validation

Metagenomic classifiers were trained on a single cohort and their performance was externally assessed on all other cohorts (off-diagonal cells in Fig. 2b), which were normalized for comparability in the same way as the training dataset. All 500 models derived from the cross-validation on the training dataset (100-times-repeated fivefold cross-validation) were applied to the hold-out dataset, and median predictions were taken from all models.

#### Leave one dataset out

Data from one cohort were set aside as an external validation set, whereas data from the remaining cohorts were pooled as a single training set on which we implemented the same procedure as above for 100-times-repeated fivefold-stratified cross-validation.

### Reporting Summary

Further information on research design is available in the Nature Research Reporting Summary linked to this article.

## Online content

Any methods, additional references, Nature Research reporting summaries, source data, extended data, supplementary information, acknowledgements, peer review information; details of author contributions and competing interests; and statements of data and code availability are available at 10.1038/s41591-022-01695-5.

## Supplementary information

Supplementary Information Supplementary Figures 1–3

Reporting Summary

Supplementary Table 1 Response rates for single agent versus combination in PRIMM-NL and PRIMM-UK.

Supplementary Table 2 Composition of plant and modified Mediterranean diet scores.

Supplementary Table 3 Meta-analysis using ORR and species relative abundances.

Supplementary Table 4 Meta-analysis using PFS12 and species relative abundances.

Supplementary Table 5 Meta-analysis of colorectal cancer datasets using species relative abundances.

Supplementary Table 6 *Blastocystis* presence in the cohorts and associations with response.

Supplementary Table 7 Meta-analysis using ORR and KO relative abundances.

Supplementary Table 8 Meta-analysis using PFS12 and KO relative abundances.

Supplementary Table 9 Meta-analysis using ORR and level 4 enzyme category relative abundances.

Supplementary Table 10 Meta-analysis using PFS12 and level 4 enzyme category relative abundances.

Supplementary Table 11 Publicly available cohorts of patients with melanoma treated with ICIs.

## Data Availability

The metagenomes and main metadata relevant to the analyses are deposited in the European Nucleotide Archive under accession number PRJEB43119. The four publicly available datasets were downloaded through the Sequence Read Archive using the accession numbers SRP197281, ERP104610, SRP116709 and SRP115355. All MetaPhlAn 3 and HUMAnN 3 profiles are available within the latest version of curatedMetagenomicData (https://bioconductor.org/packages/curatedMetagenomicData/).
