# Supplementary material for: Cross-cohort gut microbiome associations with immune checkpoint inhibitor response in advanced melanoma
Source: Nat Med. 2022 Feb 28;28(3):535–44. doi: 10.1038/s41591-022-01695-5 (PMC8938272; doi:10.1038/s41591-022-01695-5)
Supplement: Supplementary file 1 — Supplementary Figures 1–3 [file 41591_2022_1695_MOESM1_ESM.pdf]

nature portfolio

https://doi.org/10.1038/s41591-022-01695-5

Supplementary information

Cross-cohort gut microbiome associations with immune checkpoint inhibitor response in advanced melanoma

In the format provided by the authors and unedited

Supplementary Information

Dietary Score	PRIMM-UK mean score (SD)	PRIMM-NL mean score (SD)	FDR (MWW-test)
mMED	2.5 (1.11)	2.6 (1.19)	0.7022
oPDI	36.71 (3.83)	33.44 (3.99)	0.0004*
hPDI	40.63 (6.91)	43.62 (5.01)	0.0579
uPDI	39.04 (5.01)	40.07 (3.79)	0.4546

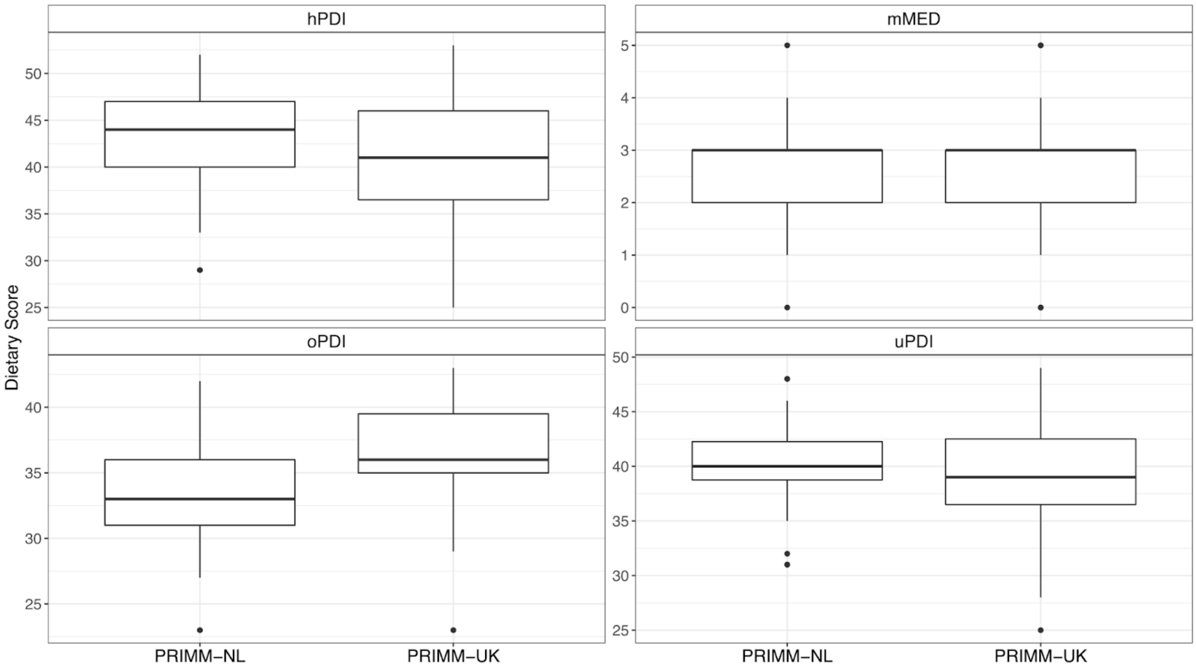

Supplementary Figure 1. Boxplots showing four food-based scores calculated to address relative dietary quality between PRIMM-NL (n = 44) and PRIMM-UK (n = 47): the modified Mediterranean diet score (mMED), original plant-based diet index (o-PDI), healthy plant-based diet index (h-PDI) and unhealthy plant-based diet index (u-PDI). The composition of scores by food groups in the UK and Dutch cohorts is given in **Supplementary Table 2**. The lower and upper hinges of boxplots correspond to the 25th and 75th percentiles, respectively. The midline is the median. The upper and lower whiskers extend from the hinges to the largest (or smallest) value no further than ×1.5 interquartile range from the hinge, defined as the distance between the 25th and 75th percentiles.

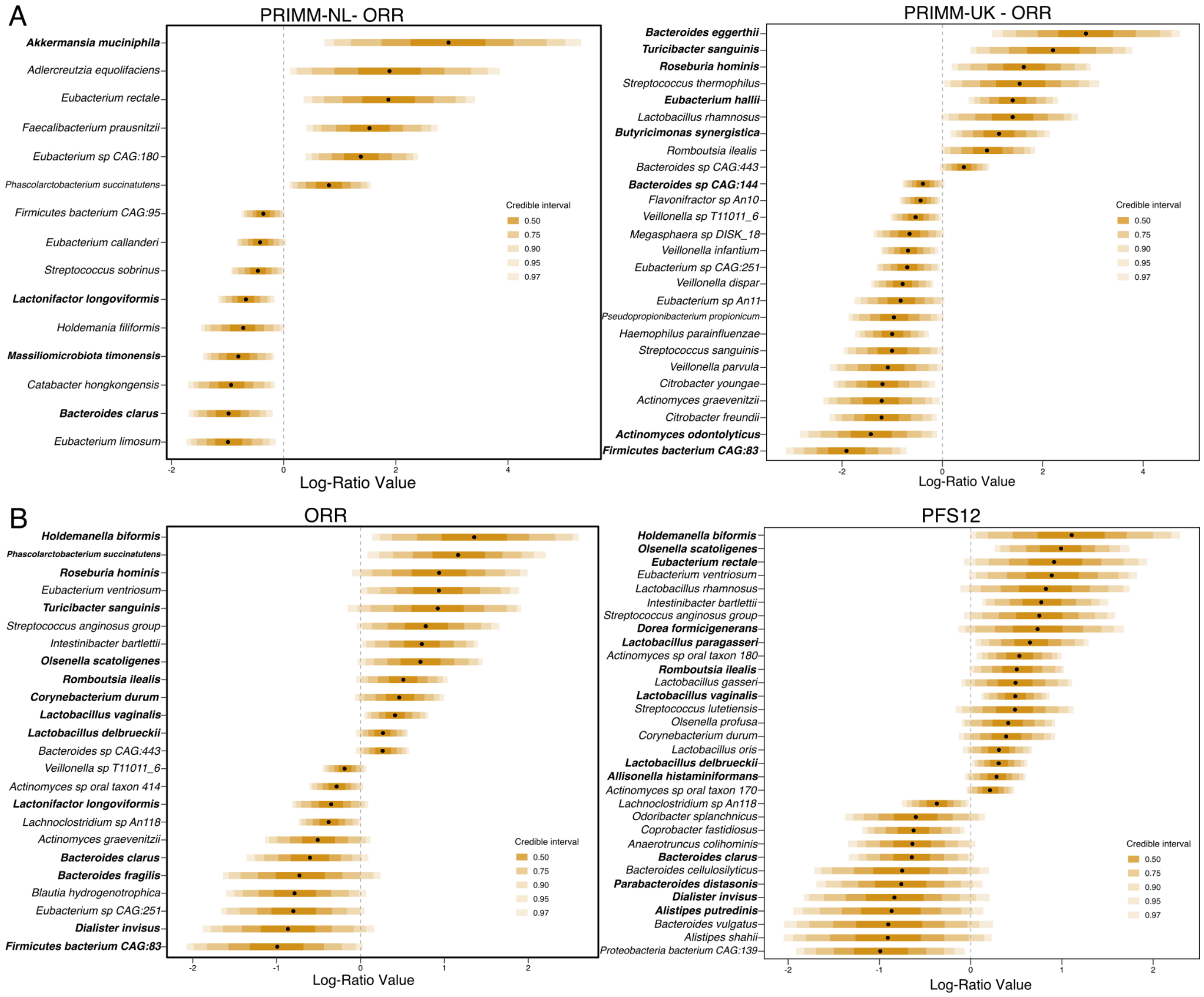

Supplementary Figure 2. Results of *pibble* models on clr transformed species-level relative abundances. (A) Species associated with ORR in the two PRIMM cohorts after adjusting for covariates that included PPI, antibiotic and steroid use, gender, performance status, previous therapy, age and ICI. **(B)** Species associated with ORR or PFS12 in the newly available cohorts adjusting for covariates that included cohort, PPI, antibiotic and steroid use, gender, performance status, previous therapy, age and ICI.

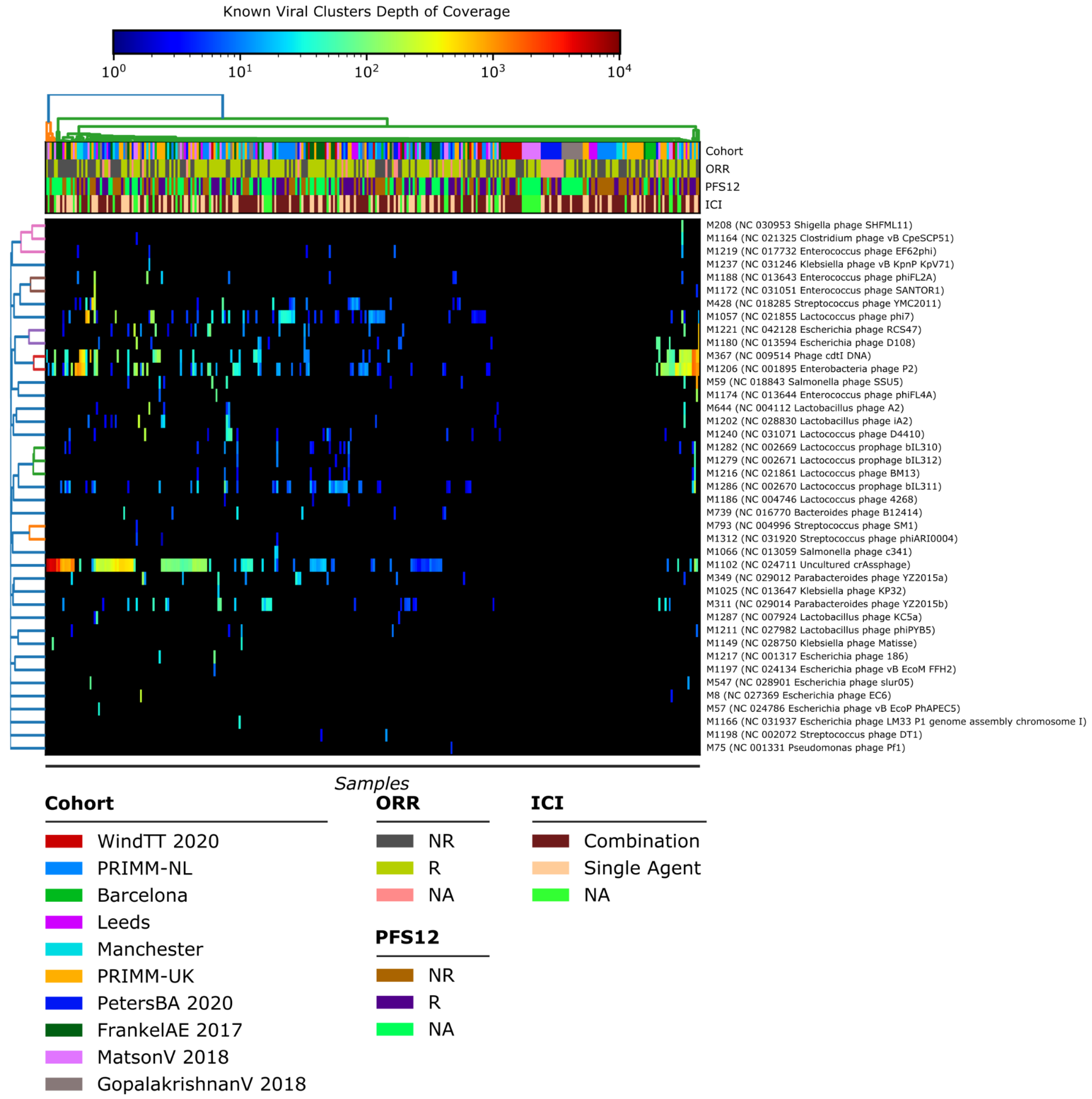

Supplementary Figure 3. Heatmap showing the depth of coverage of RefSeq viral sequences found in the cohorts. We found no significant associations with response (ORR or PFS12, q-value > 0.2) when meta-analyzing the presence/absence of these viral clusters across the different cohorts.
