# Supplementary material for: Cross-cohort gut microbiome associations with immune checkpoint inhibitor response in advanced melanoma
Source: Nat Med. 2022 Feb 28;28(3):535–44. doi: 10.1038/s41591-022-01695-5 (PMC8938272; doi:10.1038/s41591-022-01695-5)
Supplement: Supplementary file 2 — Reporting Summary [file 41591_2022_1695_MOESM2_ESM.pdf]

nature research

Corresponding author(s): Nicola Segata

Last updated by author(s): 20-12-2021

Reporting Summary

Nature Research wishes to improve the reproducibility of the work that we publish. This form provides structure for consistency and transparency in reporting. For further information on Nature Research policies, see our Editorial Policies and the Editorial Policy Checklist.

Statistics

For all statistical analyses, confirm that the following items are present in the figure legend, table legend, main text, or Methods section.

n/a	Confirmed	
☐	☒	The exact sample size (n) for each experimental group/condition, given as a discrete number and unit of measurement
☐	☒	A statement on whether measurements were taken from distinct samples or whether the same sample was measured repeatedly
☐	☒	The statistical test(s) used AND whether they are one- or two-sided<br>*Only common tests should be described solely by name; describe more complex techniques in the Methods section.*
☐	☒	A description of all covariates tested
☐	☒	A description of any assumptions or corrections, such as tests of normality and adjustment for multiple comparisons
☐	☒	A full description of the statistical parameters including central tendency (e.g. means) or other basic estimates (e.g. regression coefficient) AND variation (e.g. standard deviation) or associated estimates of uncertainty (e.g. confidence intervals)
☐	☒	For null hypothesis testing, the test statistic (e.g. F, t, r) with confidence intervals, effect sizes, degrees of freedom and P value noted<br>*Give P values as exact values whenever suitable.*
☒	☐	For Bayesian analysis, information on the choice of priors and Markov chain Monte Carlo settings
☒	☐	For hierarchical and complex designs, identification of the appropriate level for tests and full reporting of outcomes
☒	☐	Estimates of effect sizes (e.g. Cohen's d, Pearson's r), indicating how they were calculated

Our web collection on statistics for biologists contains articles on many of the points above.

Software and code

Policy information about availability of computer code

Data collection	Data from questionnaires, clinical visits and laboratory data was entered using comma delimited files and excel spreadsheets.
Data analysis	Bowtie 2 (v 2.3.4.3)<br>MetaPhlAn v.3.0 (https://github.com/biobakery/MetaPhlAn)<br>HUMAnN v.3.0 (https://github.com/biobakery/humann)<br>vegan (v2.5-7) R package<br>Surrogate Variable Analysis (v3.38) R package<br>ANCOM-BC (v.1.0.1) R package<br>MetaVolcanoR (v.1.4.0) R package<br>DESeq2 (v.1.30.0) R package<br>Zinbwave (v.1.12.0) R package<br>limma (v3.46.0) R package<br>edgeR (v.3.32.0) R package<br>Maaslin2 (v.1.4.0) R package<br>SIAMCAT (v.1.6.0) R package<br>Survival (v.3.2-7) R package<br>CMSeq (https://github.com/SegataLab/cmseq)

For manuscripts utilizing custom algorithms or software that are central to the research but not yet described in published literature, software must be made available to editors and reviewers. We strongly encourage code deposition in a community repository (e.g. GitHub). See the Nature Research guidelines for submitting code & software for further information.

Data

Policy information about availability of data

All manuscripts must include a data availability statement. This statement should provide the following information, where applicable:

- Accession codes, unique identifiers, or web links for publicly available datasets
- A list of figures that have associated raw data
- A description of any restrictions on data availability

The metagenomes and main metadata relevant to the analyses are deposited in the European Nucleotide Archive under accession no. PRJEB43119. The 4 publicly available datasets were downloaded through the Sequence Read Archive using the accession numbers SRP197281, ERP104610, SRP116709 and SRP115355. All MetaPhlAn 3 and HUMAnN 3 profiles are available within the latest version of curatedMetagenomicData (https://bioconductor.org/packages/curatedMetagenomicData/).

Field-specific reporting

Please select the one below that is the best fit for your research. If you are not sure, read the appropriate sections before making your selection.

☒ Life sciences ☐ Behavioural & social sciences ☐ Ecological, evolutionary & environmental sciences

For a reference copy of the document with all sections, see nature.com/documents/nr-reporting-summary-flat.pdf

Life sciences study design

All studies must disclose on these points even when the disclosure is negative.

Sample size

The PRIMM studies are two separate prospective observational cohort studies recruiting subjects in parallel in the U.K. (PRIMM-UK) and Netherlands (PRIMM-NL) since 2018. To study the role of the gut microbiome in ICI response, we performed shotgun metagenomic sequencing of baseline stool samples from the PRIMM cohorts, as well as three additional cohorts of ICI-naive patients with advanced cutaneous melanoma (originating from Barcelona, Leeds, and Manchester). A total of 165 microbiome samples sequenced from the patients enrolled in this study, were analyzed together with 147 samples from smaller publicly-available datasets. This provided the largest possible assessment of the potential of the gut microbiome as a biomarker of response to ICI, and allowed identification of specific microbial species or functions associated with response.

Data exclusions

We excluded samples of participants with non-metastasized and resectable Stage III melanoma who received ICI's as adjuvant treatment. Moreover patients who were not immunotherapy-naïve were excluded.

Replication

We performed a cross-cohort meta-analysis, adding three smaller cohorts and five previously published datasets with consistent metagenomic and response data to the PRIMM cohorts. The analyses showed limited reproducibility of microbiome-based response predictions across these cohorts as outlined in detail in the results and discussion (page 6 ff; page 15 ff). The limited reproducibility may in part result from heterogeneity across studies as we found a strong cohort-dependent effect in the microbial population. It is likely not attributable to analytical choices, as applying the same meta-analysis methods in the context of colorectal cancer confirmed the strong and consistent biomarkers across cohorts (Thomas et al. Nat. Med. 2019). Our study shows that the role of the gut microbiome for ICI-response is more complex than previously thought and extends beyond absence or presence of microbial species or functions in responders versus non-responders.

Randomization

n/a

Blinding

There was no control or placebo arm therefore blinding was not applicable

Reporting for specific materials, systems and methods

We require information from authors about some types of materials, experimental systems and methods used in many studies. Here, indicate whether each material, system or method listed is relevant to your study. If you are not sure if a list item applies to your research, read the appropriate section before selecting a response.

Materials & experimental systems

n/a	Involved in the study	
☒	☐	Antibodies
☒	☐	Eukaryotic cell lines
☒	☐	Palaeontology and archaeology
☒	☐	Animals and other organisms
☐	☒	Human research participants
☐	☒	Clinical data
☒	☐	Dual use research of concern

Methods

n/a	Involved in the study	
☒	☐	ChIP-seq
☒	☐	Flow cytometry
☒	☐	MRI-based neuroimaging

Human research participants

Policy information about studies involving human research participants

Population characteristics	Covariates adjusted for in the analyses included PPI, antibiotic and steroid use, gender, performance status, previous therapy, age, ICI agent (combination Ipilimumab/Nivolumab or single-agent). There were two statistically relevant clinical differences between the 2 prospective PRIMM-cohorts: the proportion of subjects who had received previous systemic targeted therapy (40% within PRIMM-NL and 20% within PRIMM-UK, P=0.03, Fisher's exact test) and the proportion with BRAF-mutated tumors (55% within PRIMM-NL and 31% within PRIMM-UK, P=0.02, Fisher's exact test, Table 1).
Recruitment	The PRIMM studies are two separate prospective observational cohort studies recruiting subjects in parallel in the U.K. (PRIMM-UK) and Netherlands (PRIMM-NL) since 2018. Patients who fulfilled the following criteria were eligible for the analyses: (i) histologically or cytologically confirmed non resectable advanced (stage III or IV) cutaneous melanoma (ii) treatment with ICI (nivolumab, pembrolizumab, ipilimumab or a combination of ipilimumab and nivolumab) at recommended dose as first-line ICI, (iii) 18 year of age or older. High quality fecal samples were collected from these patients before initiation of ICI (n=55 for UK cohort, n=55 for Dutch cohort). Additional patients were enrolled from cohorts outside the setting of a prospective clinical trial and performed in Leeds (n=19), Barcelona (n=12) and in Manchester (n=30) between March 2015 and November 2019, but from whom fecal samples were collected at similar timepoints to those collected in our included prospective studies. Written informed consent was obtained from all patients.
Ethics oversight	King's College London (KCL); Medical Ethical Committee of the University Medical Center Groningen (METc UMCG); Manchester Cancer Research Centre (MCRC) Biobank Ethics and MCRC Biobank Access Committee; Ethical committee of Hospital Clinic of Barcelona.

Note that full information on the approval of the study protocol must also be provided in the manuscript.

Clinical data

Policy information about clinical studies

All manuscripts should comply with the ICMJE guidelines for publication of clinical research and a completed CONSORT checklist must be included with all submissions.

Clinical trial registration	NCT03643289; NCT04193956; https://www.trialregister.nl/trial/7839; MCRC 07/H1003/161+5 and MCRC 13_RIMA_01; HCB/2015/1032; REC Ref 15/NW/0933.
Study protocol	https://www.clinicaltrials.gov
Data collection	The PRIMM studies are two separate prospective observational cohort studies recruiting subjects in parallel in the U.K. (PRIMM-UK) and Netherlands (PRIMM-NL) since 2018. Additional patients were enrolled from cohorts outside the setting of a prospective clinical trial and performed in Leeds (n=19), Barcelona (n=12) and in Manchester (n=30) between March 2015 and November 2019, but from whom fecal samples were collected at similar time points to those collected in our included prospective studies.
Outcomes	Clinical endpoints were defined as objective response rate (ORR) and progression free survival (PFS) at 6 and 12 months.<br><br>Response to ICI was classified according to RECIST v1.1 criteria. On the basis of radiographic response, patients were classified as Responders (CR, PR, or SD) or Non-responders (PD). In order to include late responders in our analysis, patients with progressive disease (PD) on the first radiological evaluation but a response at the second radiological evaluation compared to baseline were also labelled responders. Patients with PD on the first radiological evaluation that was confirmed on the next follow-up scan, or patients with PD on the first radiological evaluation that were unable to complete a confirmation scan due to clinical progression or death were labelled non-responders.<br><br>PFS was defined as the time from first dose of ICI to first event i.e. disease progression or death from any cause.
